# Image-based personalization of computational models for predicting response of high-grade glioma to chemoradiation

**DOI:** 10.1038/s41598-021-87887-4

**Published:** 2021-04-19

**Authors:** David A. Hormuth, Karine A. Al Feghali, Andrew M. Elliott, Thomas E. Yankeelov, Caroline Chung

**Affiliations:** 1 Oden Institute for Computational Engineering and Sciences, The University of Texas at Austin, Austin, 201 E. 24th Street, POB 4.102, 1 University Station (C0200), Austin, TX 78712-1229 USA; 2 Department of Biomedical Engineering, The University of Texas at Austin, Austin, Austin, TX USA; 3 Department of Diagnostic Medicine, The University of Texas at Austin, Austin, Austin, TX USA; 4 Department of Oncology, The University of Texas at Austin, Austin, Austin, TX USA; 5 Livestrong Cancer Institutes, The University of Texas at Austin, Austin, Austin, TX USA; 6 Department of Imaging Physics, MD Anderson Cancer Center, Houston, TX USA; 7 Department of Radiation Oncology, MD Anderson Cancer Center, Houston, TX USA

**Keywords:** Computational models, Cancer imaging, CNS cancer, Biomedical engineering

## Abstract

High-grade gliomas are an aggressive and invasive malignancy which are susceptible to treatment resistance due to heterogeneity in intratumoral properties such as cell proliferation and density and perfusion. Non-invasive imaging approaches can measure these properties, which can then be used to calibrate patient-specific mathematical models of tumor growth and response. We employed multiparametric magnetic resonance imaging (MRI) to identify tumor extent (via contrast-enhanced *T*_*1*_*-*weighted, and *T*_*2*_-FLAIR) and capture intratumoral heterogeneity in cell density (via diffusion-weighted imaging) to calibrate a family of mathematical models of chemoradiation response in nine patients with unresected or partially resected disease. The calibrated model parameters were used to forecast spatially-mapped individual tumor response at future imaging visits. We then employed the Akaike information criteria to select the most parsimonious member from the family, a novel two-species model describing the enhancing and non-enhancing components of the tumor. Using this model, we achieved low error in predictions of the enhancing volume (median: − 2.5%, interquartile range: 10.0%) and a strong correlation in total cell count (Kendall correlation coefficient 0.79) at 3-months post-treatment. These preliminary results demonstrate the plausibility of using multiparametric MRI data to inform spatially-informative, biologically-based predictive models of tumor response in the setting of clinical high-grade gliomas.

## Introduction

High-grade gliomas are aggressive, infiltrative and heterogenous malignancies that despite current combinatorial therapy with aggressive surgery followed by adjuvant radiotherapy (RT) and chemotherapy (CT) are highly likely to recur or progress in the brain^1^. The wide range of treatment responses across patients following our current surgical and conventional RT and CT regimens used in clinical practice both supports and informs the physiological and biological heterogeneity that has been recognized across individual tumors^2,3^. An approach to increasing treatment efficacy has been the use of patient-specific data including the application of personalized targeting of highly conformal RT; however, a promising advance in this approach is using biologically-guided treatment that targets areas of anticipated tumor progression and treatment resistance^4,5^. Current assessment of patient-specific response to therapy whether in clinical practice or even with clinical trials using Radiological Assessment in Neuro-Oncology (RANO^6^) criteria is dependent on the monitoring of radiological and clinical changes over weeks to months after completing a course of treatment before determining tumor progression, ultimately delaying the cessation of ineffective treatments for a potentially effective one thereby impacting patient survival, functional status, and quality of life.

If disease progression could be determined with greater confidence at the first signs of tumor progression or even *predicted*, rather than assessed, on an individual patient basis, treatment plans could be adapted to prevent or impede disease progression. Promising developments in the field of mathematical oncology^7^ have generated experimental and computational approaches to characterize^8–12^ and predict future tumor growth and response^13–17^ for individual tumors. In particular, biologically-based mathematical models (as opposed to statistical models) of tumor growth and response to therapy, which are calibrated or personalized for individual patients, have the potential to provide clinicians with actionable “forecasts”^18,19^ to improve outcomes.

There is a rich literature in mathematical modeling of gliomas^20–22^ on topics from, but not limited to, resection planning^23^, response to RT^16,24,25^, angiogenesis^26,27^, and mass effect^11,28^. In particular, much progress has been made using in vivo imaging data to initialize and constrain these models^21^. One promising approach by Neal et al.^10^ combined anatomical/structural imaging data with tumor growth simulations to devise a novel “Days Gained” response metric to assess treatment response in patients with high-grade gliomas. In 33 patients the “Days Gained” metric was able to identify those who would have improved overall survival. While Neal et al.’s approach provides a metric for identifying patients who may have a worse prognosis, it does not provide a spatial-map to locate where or which subregions of disease may be resistant to the current therapy and ultimately is likely to progress. A map of spatial response could assist localized treatment planning to target less responsive disease. To this end, our approach^16,27^ leverages the use of anatomical/structural *and* quantitative magnetic resonance imaging (MRI) to calibrate predictive models of spatial response and growth. Incorporating quantitative, biologically-sensitive, imaging measures such as diffusion-weighted imaging (DWI) with anatomical/structural imaging enables voxel-wise forecasts of treatment response. In the present contribution, we use MRI data sensitive to the extent of tumor burden, and cell density (via DWI) to calibrate model parameters on a patient-specific basis, thereby enabling patient-specific predictions without the need of a large training data set^29^. The extent of disease in this model is determined by both contrast-enhanced (CE-) *T*_*1*_-MRI and *T*_*2*_-FLAIR (fluid-attenuated inversion recovery) MRI^29^. Enhancement observed on CE-MRI indicates the local breakdown of the blood tumor barrier commonly associated with high-grade tumors, whereas the hyperintense signal abnormalities in *T*_*2*_-FLAIR MRI signifies a mixture of vasogenic edema, infiltrative edema, and infiltrative tumor cells^30^. The tumor cellularity is estimated via DWI in which a set of diffusion-weighted images are collected in order to measure the apparent diffusion coefficient (or *ADC*) of water within tissue. In well-controlled scenarios, it has been shown that as cell density increases, water mobility and the *ADC* decreases^31,32^. DWI has shown promise as an early imaging biomarker for response in high-grade gliomas^33^, and is used widely throughout other areas of oncology and RT^34,35^. By using both anatomical and quantitative MRI techniques we are able to predict spatiotemporal changes in both the volumetric and intratumoral cellularity characteristics^14^.

Here we present a novel approach to forecast the spatial response to chemoradiation using patient-calibrated mathematical models. We have developed a family of biologically-based mathematical models of tumor growth and response to chemoradiation built upon the standard reaction–diffusion model of tumor growth^36^. Within this family of models, we investigate ten approaches to spatially couple patient imaging data and treatment efficacy. Additionally, we have developed a two-species model of tumor growth (similar to the approach of Gatenby et al.^37^) and response describing the spatio-temporal evolution of the enhancing and the non-enhancing clinical tumor volume regions. Each model is then calibrated to each individual patient dataset (collected during standard-of-care imaging visits) resulting in a set of patient-specific growth and response model parameters. We then use model selection to identify the most parsimonious model that best balances model fit and complexity (i.e., number of parameters) for each patient. We then evaluate model performance by calculating the error in model fits and predictions to MRI observations obtained at future time points at the global (i.e., volumetric) and local (i.e., voxel) levels.

## Methods

### Patient cohort

Nine patients with histologically confirmed high-grade gliomas were included in this study under a protocol approved by the institutional review board at M.D. Anderson Cancer Center. An informed consent waiver was obtained from the institutional review board at the M.D. Anderson Cancer Center for this retrospective study. All methods were performed in accordance with relevant guidelines and regulations. These patients had unresected or partially resected disease followed by standard-of-care treatment and MR imaging acquired at least at baseline, 1-month, and 3-months following treatment at at the M.D. Anderson Cancer Center. The exact dates varied across patients by up to 0.5 month; however, due to a small patient cohort the imaging visits were grouped into 1-, 3-, and 5-month visits for analysis purposes. Table 1 summarizes the clinical features of these patients. Each patient received radiotherapy to a total of 60 Gy (Gy) delivered in 2 Gy per fraction per weekday for 6 weeks, concurrently with temozolomide 75 mg/kg delivered orally 7 days per week^1^. Adjuvant chemotherapy consisted of at least six cycles of temozolomide 150–200 mg/kg delivered orally for 5 days during each 28-day cycle^1^. For each patient, we included all available imaging time points until disease progression was identified (as assessed after 12 weeks post-radiotherapy^6^) or when records indicated they switched treatment protocols.

**Table 1:** Clinicopathologic characteristics of the patient cohort.

Patient	Age (years)	Sex	Tumor location	Imaging visits
1	49	F	Left frontal lobe	B, 1 m, 3 m
2	61	M	Right parietal lobe	B, 1 m, 3 m, 5 m
3	54	F	Left frontal lobe	B, 1 m, 3 m, 5 m
4	55	M	Right thalamus/posterior basal ganglia	B, 1 m, 3 m
5	68	M	Right temporal lobe	B, 1 m, 3 m, 5 m
6	59	M	Right frontal lobe	B, 1 m, 3 m
7	53	M	Left frontal lobe	B, 1 m, 3 m
8	50	M	Body of the corpus callosum	B, 1 m, 3 m
9	63	F	Left frontal lobe	B, 1 m, 3 m, 5 m

### MRI data and processing

**Figure 1:**
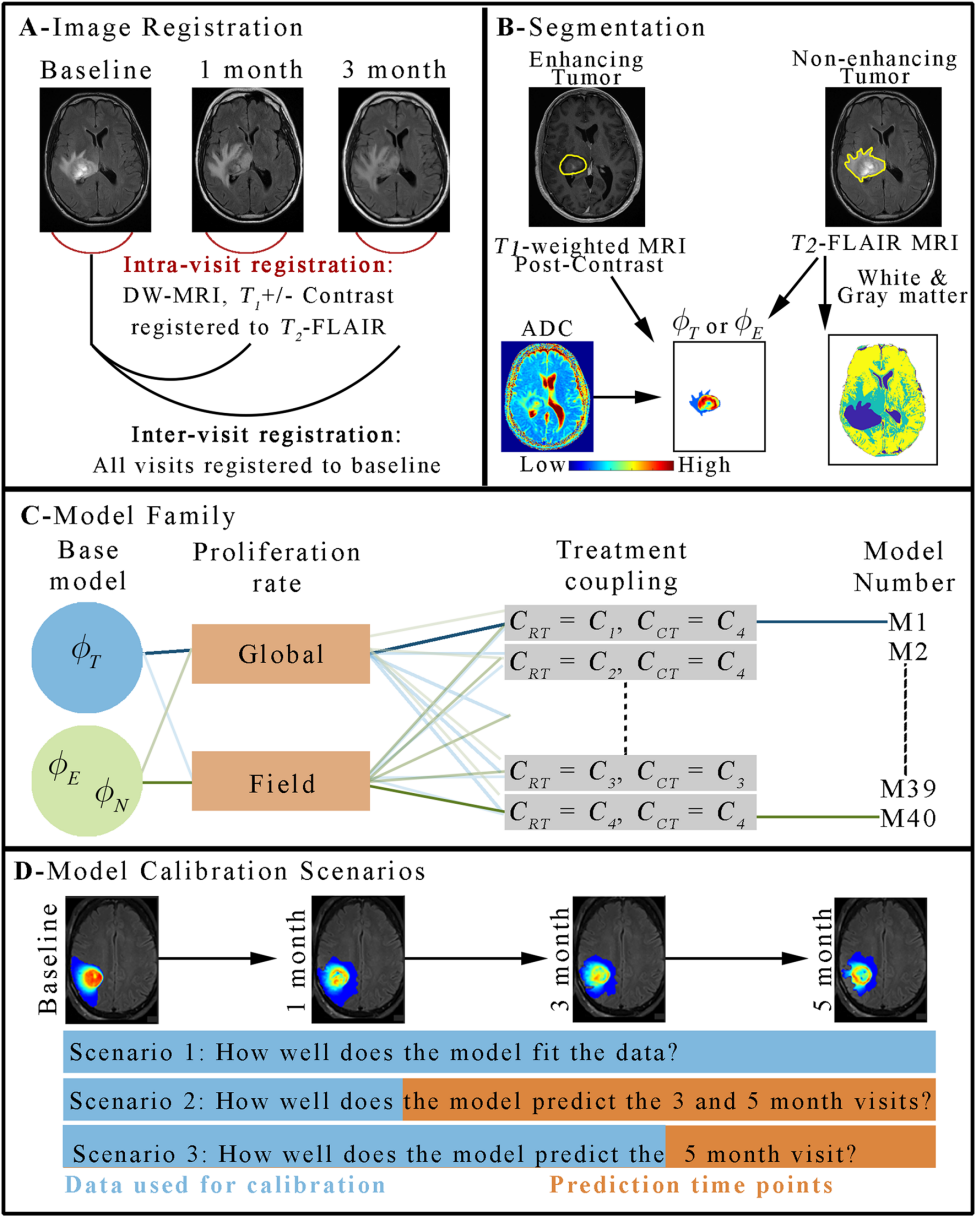
Schematic of the image processing and computational methods. Panel (**A**) shows the approach to image registration where all images first receive an intra-visit registration to align all images within a single visit prior to an inter-visit registration to the baseline time point. Panel (**B**) shows the expertly segmented contrast-enhancing and non-enhancing (*T*_*2*_ hyperintense) tumor regions which are used as ground truth of enhancing tumor and non-enhancing clinical tumor volumes, respectively. DWI measurements of ADC are then used to estimate *ϕ*_*T*_ and *ϕ*_*E*_, while *ϕ*_*N*_ is set to a fixed value within the clinical tumor volume. The *T*_*2*_-FLAIR image is also used to provide a segmentation of white, gray, and cerebral spinal fluid tissues. Panel (**C**) shows our framework for constructing models. A family of models are built upon either a single-species (*ϕ*_*T*_) or two-species (*ϕ*_*E*_ and *ϕ*_*N*_) reaction–diffusion model. The proliferation rate is either assigned globally or as a field, and there are 10 approaches to spatially vary the efficacy of RT and CT resulting for a total of 40 models. Finally, panel (**D**) shows the three calibration scenarios to evaluate model fits, long-term model “forecasts”, and short-term model “forecasts”, respectively. MATLAB R2019b (Mathworks, Natick, MA) was used for producing individual figures, images, and graphs. Adobe Photoshop 2020 (Adobe, San Jose, CA) was used to arrange individual panels, draw schematics, and add text.

We used data from four MRI sequences acquired at each scan session in our analysis: (1) a pre-contrast *T*_*1*_-weighted image, (2) a post-contrast *T*_*1*_-weighted image, (3) *T*_*2*_-FLAIR, (4) DWI. We present the salient details for image analysis and processing here, while a more complete description is found in the Supplemental Material S1. First, a rigid registration algorithm was used to register all images to the baseline *T*_*2*_-FLAIR image (Panel A in Fig. 1). For each patient visit, the enhancing tumor volume and the non-enhancing clinical tumor volume (defined as the non-enhancing, *T*_*2*_-hyperintense region) were segmented using a semi-automated approach from the post-contrast *T*_*1*_-weighted and *T*_*2*_-FLAIR images, respectively. The semi-automated approach consisted of thresholding methods in combination with manual adjustments by a radiation oncologist and secondary quality review by a second senior radiation oncologist. A *k*-means clustering of signal intensity was used to segment the white matter, gray matter, and cerebrospinal fluid from *T*_*2*_-FLAIR images^38^ (Panel B in Fig. 1).

The *ADC* calculated from DWI data was used to estimate the tumor cell volume fraction at each imaging visit using Eq. (1) as described in^13,15,39,40^:

1 $$\phi_{T} (\overline{x},t) = \left( {\frac{{ADC_{w} - ADC(\overline{x},t)}}{{ADC_{w} - ADC_{\min } }}} \right),$$

where $\phi_{T} \left( {\overline{x},t} \right)$ is the tumor volume fraction at 3D position $\overline{x}$ and time *t*, *ADC*_*w*_ is the *ADC* of free water^41^, and *ADC*_*min*_ is the minimum ADC measured. We have used this approximation previously to provide non-invasive estimates of tumor cellularity^15,17,39,40,42^; however, we note that this approximation is a simplification of all the biological aspects that contribute to changes in *ADC*. This point is discussed further in^15,27^. Within the enhancing tumor volume, we assumed that the primary cellular contribution is from tumor cells, therefore $\phi_{T} \left( {\overline{x},t} \right)$ was calculated using Eq. (1). However, within the non-enhancing clinical tumor volume the cell density or relationship to imaging features is less clear, thus we used a fixed value of 0.16^23^ everywhere within that region. An alternative approach for assigning celluarity in the non-enhancing regions (used in^10,23^ and elsewhere) is to assume a spatially varying value of cellularity decreasing from the value observed at the interface of the enhancing region to a fixed value at the periphery of the non-enhancing region. For the two-species model, the tumor volume fraction within the enhancing tumor volume was calculated using Eq. (1) and set to zero outside the enhancing tumor volume, while in the non-enhancing clinical tumor volume region, it was set to a fixed value of 0.16.

### Mechanically-coupled model of tumor growth

We have developed a family of models built upon the well-studied reaction–diffusion model that has been applied extensively to pre-clinical^13–16,27,43^ and clinical models^9,44^ of glioma growth. Panel C of Fig. 1 displays the framework for our model building process. The first set of tumor growth models, are built upon a single species version of the reaction–diffusion model, shown in Eq. (2), which describes the spatial and temporal change in tumor cell number due to the outward movement (i.e., the diffusion term) and due to the proliferation (i.e., the logistic growth term) of tumor cells:

2 $$\frac{{\partial \phi_{T} (\overline{x},t)}}{\partial t} = \overbrace {{\nabla .(D_{T} (\overline{x},t)\nabla \phi_{T} (\overline{x},t))}}^{Diffusion} + \overbrace {{k_{p,T} \phi_{T} (\overline{x},t)(1 - \phi_{T} (\overline{x},t)/\theta_{T} )}}^{Logistic\,\,\,Growth},$$

where $D_{T} \left( {\overline{x},t} \right)$ is the tumor cell diffusion coefficient, *k*_*p,T*_ is the tumor cell proliferation rate, and $\theta_{T}$ is the tumor cell carrying capacity (i.e., the maximum packing fraction that a voxel can functionally support). As it is well known that local tissue stress can inhibit tumor expansion^45^, we have incorporated this phenomena into our reaction–diffusion model. Thus, tumor cell diffusion is assumed to change spatially and temporally as a function of local tissue mechanical properties as detailed in^14,28,46^. The local tissue stress, summarized by the von Mises stress, $\sigma_{vm} (\overline{x},t)$, is used to exponentially dampen $D_{T} \left( {\overline{x},t} \right)$ according to:

3 $$D_{T} (\overline{x},t) = D_{T,0} \left( {\overline{x}} \right)\exp \left( { - \lambda_{1} \cdot \sigma_{vm} (\overline{x},t)} \right),$$

where *D*_*T,0*_ represents the uninhibited tumor cell diffusion coefficient, and $\lambda_{1}$ is the stress-tumor cell diffusion coupling constant. We assume *D*_*T,0*_ is spatially-resolved in that it can take on one value for white matter (*D*_*T,w*_), and another for gray matter (*D*_*T,g*_). We present the salient details for the implementation of this mechanically-coupled model here, while the complete numerical details are described elsewhere^46^. During each iteration, the local $\sigma_{vm} (\overline{x},t)$ is determined by solving for tissue displacement, $\vec{u}$, assuming linear elastic, isotropic equilibrium:

4 $$\nabla \cdot G\nabla \vec{u} + \nabla \frac{G}{1 - 2v}\left( {\nabla \cdot \vec{u}} \right) - \lambda_{2} \nabla \phi_{T} (\overline{x},t) = 0,$$

where *G* is the shear modulus, *υ* is Poisson’s ratio, and $\lambda_{2}$ is the second coupling constant (assigned to 1). Literature values are used to assign tissue specific *G* and *v* for white matter and gray matter^47^.

The second set of tumor growth models we have developed is a two-species reaction–diffusion model describing the evolution of the contrast-enhancing tumor region (i.e., enahncing tumor volume) and the non-enhancing, *T*_*2*_-hyperintense region (i.e., non-enhancing clinical tumor volume) described by Eqs. (5) and (6):

5 $$\frac{{\partial \phi_{E} (\overline{x},t)}}{\partial t} = \overbrace {{\nabla .(D_{E} (\overline{x},t)\nabla \phi_{E} (\overline{x},t))}}^{Diffusion} + \overbrace {{k_{p,E} \phi_{E} (\overline{x},t)(1 - (\phi_{E} (\overline{x},t) + \beta_{NE} \phi_{N} (\overline{x},t))/\theta_{E} )}}^{Logistic\,\,\,Growth},$$

6 $$\frac{{\partial \phi_{N} (\overline{x},t)}}{\partial t} = \overbrace {{\nabla .(D_{N} (\overline{x},t)\nabla \phi_{N} (\overline{x},t))}}^{Diffusion} + \overbrace {{k_{p,N} \phi_{N} (\overline{x},t)(1 - (\phi_{N} (\overline{x},t) + \beta_{EN} \phi_{E} (\overline{x},t))/\theta_{N} )}}^{Logistic\,\,\,Growth},$$

where *ϕ*_*E*_ is the volume fraction of the enhancing tumor region, *ϕ*_*N*_ is the volume fraction for the invasive non-enhancing tumor region, *β*_*NE*_ and *β*_*EN*_ are competition terms between the two regions, *D*_*E*_ and *D*_*N*_ are the diffusion coefficients, *k*_*p,E*_ and *k*_*P,N*_ are the proliferation rates, and *θ*_*E*_ and *θ*_*N*_ are the carrying capacities for the enhancing and non-ehancing regions, respectively. This two-species model represents an extension from the Gatenby et al.^48^ model for tumor and healthy cells; however, here we assume both *ϕ*_*E*_ and *ϕ*_*N*_ are tumor cells with distinct tumor growth properties. It has been shown that this non-enhancing, peritumoral region represents diffuse disease that is typically more proliferative or invasive than cells found within the enhancing region^49–51^.

### Modeling response to chemoradiation

We describe the response of tumor cells to radiation and chemotherapy as an immediate reduction of $\phi_{T} \left( {\overline{x},t} \right)$ at the time of treatment using Eq. (7):

7 $$\phi_{{T_{post} }} \left( {\overline{x},t} \right) = \phi_{T,pre} \left( {\overline{x},t} \right)SF_{RT} \left( {\overline{x},t} \right)SF_{CT} \left( {\overline{x},t} \right),$$

where $\phi_{T,post} \left( {\overline{x},t} \right)$ is the post-treatment value of the tumor cell fraction, $\phi_{T,pre} \left( {\overline{x},t} \right)$ is the pre-treatment value of the tumor cell fraction, $SF_{RT} \left( {\overline{x},t} \right)$ is the surviving fraction of tumor cells following a single dose of RT, and $SF_{CT} \left( {\overline{x},t} \right)$ is the surviving fraction of tumor cells following a single dose of CT. Similar versions of Eq. (7) are used for *ϕ*_*E*_ and *ϕ*_*N*_. *SF*_*RT*_ and *SF*_*CT*_ are piecewise functions equal to 1 when *t* is not equal to the treatment time, and between 0 and 1 otherwise. Clinical notes on the timing of the beginning and end of RT were used to define the in silico treatment times for RT and CT unique to each patient. RT and CT are assumed to occur over a single simulation time step on the day of RT and/or CT.

While the underlying mechanisms of temozolomide leading to G2M-phase arrest would sensitize tumor cells to DNA damage induced by RT, we assume both the RT and CT have independent cytotoxic effects, and do not explicitly include a synergistic effect between the CT agent and RT response in this initial approach. $SF_{RT} \left( {\overline{x},t} \right)$ and $SF_{CT} \left( {\overline{x},t} \right)$ are forumalated as one of four coupling approaches (*C*_*1*_ to *C*_*4*_) to spatially vary the efficacy of RT and CT between a minimum SF (*SF*_*RT*,min_ and *SF*_*CT,min*_) and 1. For approach *C*_*1*_, we assume that the efficacy of a given treatment *i* decreases as $\phi_{T} \left( {\overline{x},t} \right)$ approaches $\theta_{T}$ which will reduce the overall proliferation rate, and therefore make the cells less susceptible to treatment:

8 $$SF_{i} \left( {\overline{x},t} \right) = SF_{i,\min } + \left( {1 - SF_{i,\min } } \right)\left( {1 - \frac{{\phi_{T} \left( {\overline{x},t} \right)}}{{\theta_{T} }}} \right),$$

where $SF_{i} \left( {\overline{x},t} \right)$ is the surviving fraction for treatment *i* (either RT or CT), and *SF*_*i,*min_ is the minimum surviving fraction for treatment *i.* For approach *C*_*2*_, we assume the efficacy of a given treatment *i* decreases in areas that are poorly perfused using:

9 $$SF_{i} \left( {\overline{x},t} \right) = SF_{i,\min } + \left( {\frac{{1 - SF_{i,\min } }}{0.5}} \right)\left( {ER^{ - 1} - 0.5} \right),$$

where *ER* is the enhancement ratio of the post-contrast *T*_*1*_-weighted image to the pre-contrast *T*_*1*_-weighted bound between 1 and 2. Thus, as *ER* increases and approaches 2, the efficacy of treatment approaches *SF*_*i,*min_*.* Approach *C*_*3*_, is a variation on the *C*_*2*_, where we still assume the efficacy of a given treatment *i* is related to tissue perfusion using:

10 $$SF_{i} \left( {\overline{x},t} \right) = SF_{i,\min } + \left( {\frac{{1 - SF_{i,\min } }}{0.5}} \right)\left( {1 - \frac{ER}{2}} \right),$$

For approach *C*_*4*_, we assume the effects of a given treatment *i* are uniform throughout the tumor: thus $SF_{i} \left( {\overline{x},t} \right)$ = *SF*_*i,min*_.

We note that there are potentially other suitable candidates for spatially-varying the efficacy of RT and CT, such as relating effiacy to tissue oxygenation^8^ or pharmacokinetic parameters^17^. However, in this manuscript we limited the candidates to two properties which we can assign from the available data: cell density (which has been established previously^52,53^) and the enhancement ratio (which serves as surrogate for tissue perfusion).

We evaluated 10 different combinations of approaches *C*_*1*_ to *C*_*4*_ for both CT and RT. Combinations 1–3 correspond to *C*_*1*_ to *C*_*3*_ being applied only to the RT term, while *C*_*4*_ was used for CT. Combinations 4–6 correspond to *C*_*1*_ to *C*_*3*_ being applied only to the CT term, while *C*_*4*_ was used for RT. Combinations 7–10 correspond to *C*_*1*_ to *C*_*4*_ being applied to both the CT and RT terms. Supplemental Table S1 lists each model combination. Panel C in Fig. 1 shows an example of this model building process.

The spatial–temporal evolution of $\phi_{T} \left( {\overline{x},t} \right)$ was determined using a 3D finite difference approximation implemented in MATLAB R2019b (Mathworks, Natick, MA). Finite difference simulations were performed on a domain discretized in a fashion to identically match the imaging domain. This resulted in isotropic discretization in-plane, and large spatial steps in the slice direction. No refinement of the discretized simulation domain was performed at the boundaries of tissues or the skull. This faciliated a direct mapping between modeled and measured estimates of tumor growth. Domain discretization is performed on the baseline images. Additional details are presented in the Supplemental Material and for a complete description of the numerical implementation of these techinques, the interested reader is referred to^46^.

### Model parameter calibration and selection

A total of 40 models were developed from two base models, 10 therapy coupling combinations, and two proliferation parameterization approaches (i.e., *k*_*p,T*_ and *k*_*p,E*_ assigned as uniform or field within tumor). The remaining calibrated model parameters (in Table 2) were fit as a global variable. We considered three different calibration/prediction scenarios (Fig. 1D). For the first scenario, we calibrated each model to all of the available data to see how well the models describe that data. For the second and third scenarios, we calibrated each model to a subset of the available data and then those calibrated parameters are used to run the model forward in time to predict the tumor response at that patient’s remaining imaging visits.

**Table 2:** Model parameters and variables.

Parameter or variable	Definition	Source
$\phi_{T} \left( {\overline{x},t} \right)$	Tumor cell volume fraction	Measured from DWI
$\phi_{E} \left( {\overline{x},t} \right)$	Enhancing tumor volume fraction	Measured from DWI
$\phi_{N} \left( {\overline{x},t} \right)$	Non-enhancing tumor volume fraction	Assigned to fixed value
*k*_*p,T,*_* k*_*p,E*_* k*_*p,N*_	Proliferation rates	Calibrated
*β*_*NE*_, *β*_*EN*_	Competition terms	Set to 4 and 1
*θ*_*T,*_*, θ*_*E,*_*, θ*_*N*_	Carrying capacity	Calibrated, *θ*_*N*_ set to 0.16
*D*_*T,w,*_* D*_*E,w*_, *D*_*N,w*_, *D*_*T,g,*_* D*_*E,g*_, *D*_*N,g*_	Diffusion coefficients in absence of mechanically coupling for white and gray matter	Calibrated
*G*_*w*_	Shear modulus for white matter	2.7 kPa^47^
*G*_*g*_	Shear modulus for gray matter	3.1 kPa^47^
*V*	Poisson’s ratio	Set to 0.45
λ_*1*_	Coupling constants	Calibrated
λ_*2*_	Coupling constants	Set to 1
*SF*_*RT,min*_	Surviving fraction following radiotherapy	Calibrated
*SF*_*CT,min*_	Surviving fraction following chemotherapy	Calibrated

**Figure 2:**
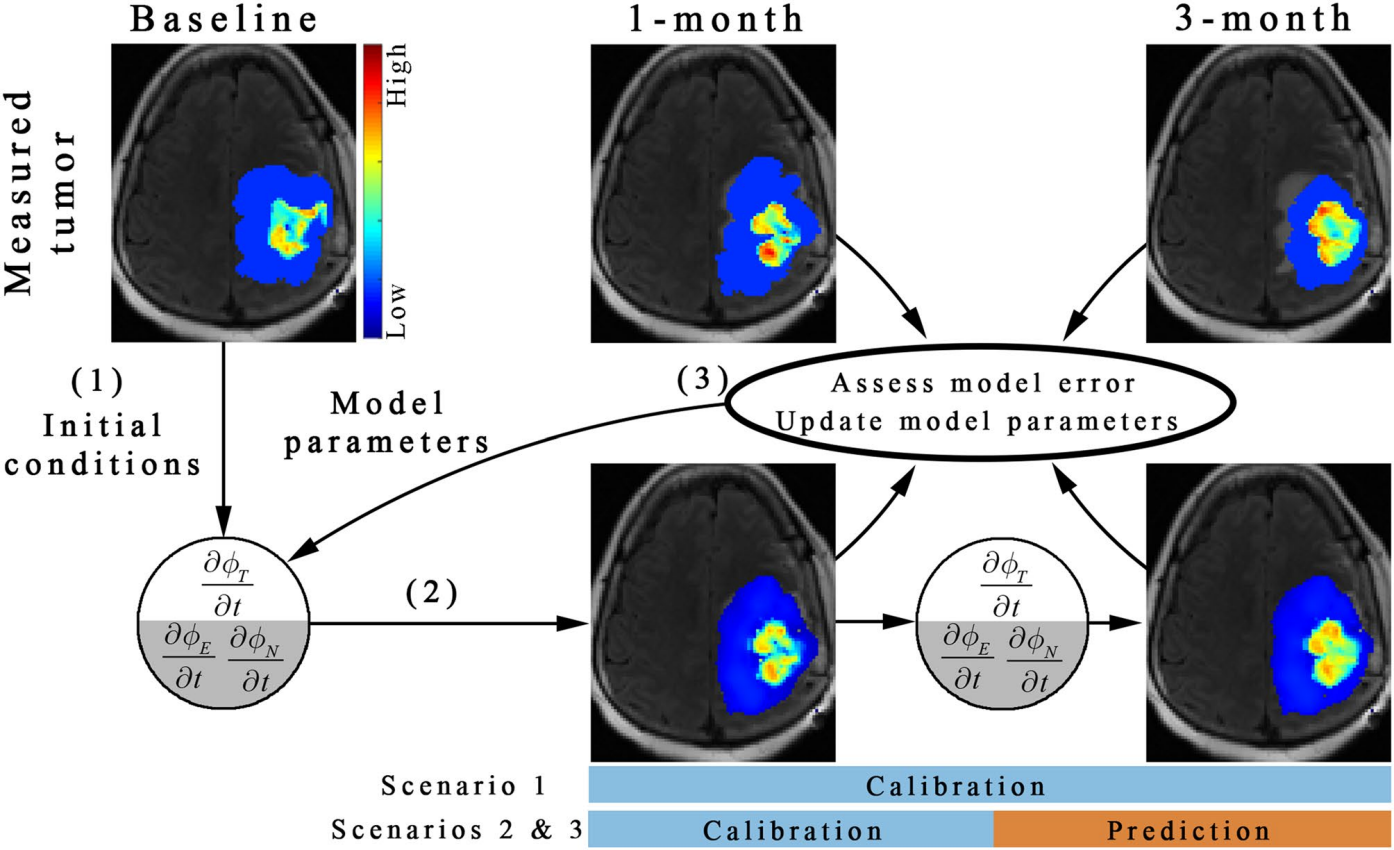
Schematic of the model calibration and prediction framework. The central slice from patient 1 is used to illustrate the model calibration and prediction framework. First, the baseline tumor distribution and an initial guess of model parameters are used in a forward run of the of either the single species (*ϕ*_*T*_) or the two species (*ϕ*_*E*_ and *ϕ*_*N*_) model to estimate the spatial distribution of tumor cells. Second, the predicted tumor distribution is sampled at the 1-month and 3-month time points. Third, the error between the measured and the model predicted tumor distribution is computed and then used to update model parameters (using the Levenberg–Marquardt approach). This model calibration process continues until stopping criterion are met (e.g., iteration count, or convergence of objective function). For scenario 1, all available imaging time points (i.e., 1-month and 3-month for patient 1) are used to calibrate the model parameters. For scenarios 2 and 3, a subset of the imaging time points (e.g., only 1-month) are used to calibrate model parameters leaving the remaining imaging time points to evaluate prediction accuracy. MATLAB R2019b (Mathworks, Natick, MA) was used for producing individual figures, images, and graphs. Adobe Photoshop 2020 (Adobe, San Jose, CA) was used to arrange individual panels, draw schematics, and add text.

We used the Levenberg–Marquardt^46,54^ algorithm to minimize errors between the measured and simulated tumor growth. An initial guess of model parameters and baseline initial conditions (arrow 1 in Fig. 2) are used in a finite difference simulation for a given model. The finite difference simulation is then sampled at the imaging visits used for calibration (arrow 2 in Fig. 2) and the error is assessed between the model and the measurement. The residual error is used within the algorithm to update model parameters (arrow 3 in Fig. 2). For the prediction scenarios, the calibrated parameters were then used to run the model forward in time to predict tumor growth at the remaining time points not used for model calibration. Complete technical details on the model calibration can be found in the Supplemental Materials S1 and in^46^. Additionally, an analysis of the robustness of parameter estimation to measurement noise is reported in Supplemental Table S3 which was observed to be less than 5.6% error in parameter estimates when the baseline and 1-month image are used for model calibration.

The Akaike Information Criterion (*AIC*^55^) was used to select the model that balances model complexity and model-data agreement. We calculated the *AIC* for each model over the timepoints used for model calibration. The two species model, with a locally varying proliferation rate and radiation and chemotherapy both coupled to approach C2 (i.e., coupled to *ER*) was selected as the model with the lowest average *AIC* across all patients (see Supplemental Table S4 for complete results). This model will be used in all of the model calibrations and predictions reported in the results. Complete technical details on model selection are presented in the supplemental materials S1.

### Error analysis

The error between the model and measured tumor growth was assessed at the global (i.e., general size and overlap) and local (i.e., voxel-wise agreement) levels. At the global level, we calculated the percent error in predicted tumor volume and the degree of overlap with the Dice coefficient. A Dice value of 1 indicates a perfect overlap, whereas a Dice value of 0 indicates no overlap in the predicted and observed tumor volumes. For the two-species model we also calculated the percent error and Dice values individually for the non-enhancing and enhancing regions. At the local level, we calculated the concordance correlation coefficient (CCC) and the Pearson correlation coefficient (PCC) to assess the level of agreement and correlation between the predicted and measured values at each voxel location. Due to small sample size, we used non-parametric approaches such as the box plot and the Kendall rank correlation coefficient (KCC) to report summary statistics.

## Results

### Scenario 1: evaluation of model calibration

**Figure 3:**
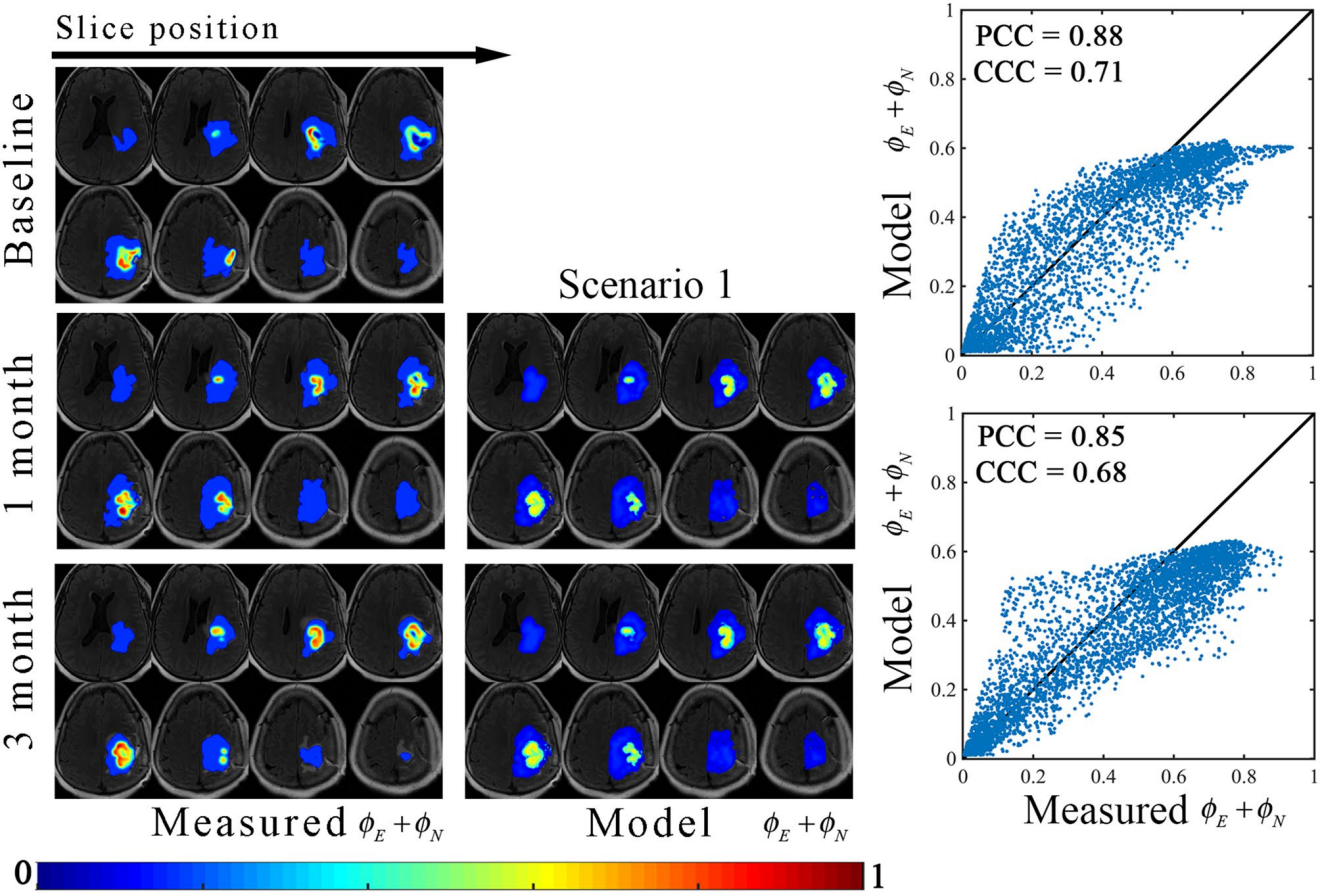
Representative results from scenario 1. The central eight tumor slices from patient 1 are used to demonstrate the model calibration results using the best model as determined by the AIC. The measured and model determined sum of the enhancing and non-enhancing regions (i.e., *ϕ*_*E*_ and *ϕ*_*N*_) are shown in left and middle columns, respectively, whereas the voxel-wise comparison between the model and measured tumor distributions are shown in the right column. In general, a high level of agreement and correlation resulted in low voxel level errors (CCCs greater than 0.68). MATLAB R2019b (Mathworks, Natick, MA) was used for producing individual figures, images, and graphs. Adobe Photoshop 2020 (Adobe, San Jose, CA) was used to arrange individual panels, draw schematics, and add text.

**Figure 4:**
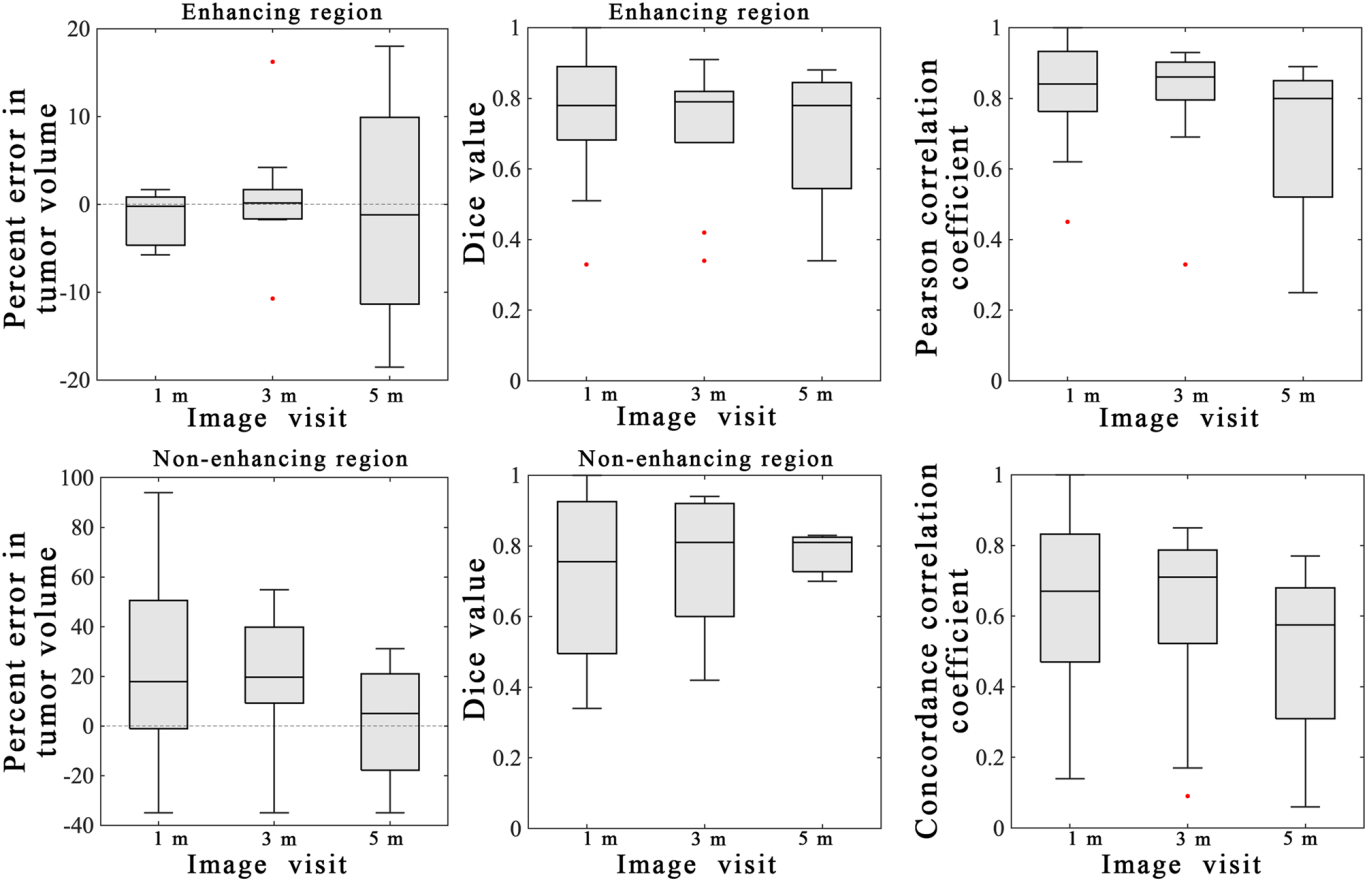
Summary statistics from scenario 1. Error analysis from the model calibration scenario are shown for the entire cohort using the best model. The left column shows the percent error in the tumor volume for the enhancing (top) and non-enhancing (bottom) regions. Greater error was observed in the non-enhancing regions versus the enhancing regions. The middle column shows the Dice values for the enhancing (top) and non-enhancing (bottom) regions. A high level of overlap (median Dice values all greater than 0.76) were observed for the enhancing regions and the non-enhancing regions. The third column shows the results of the voxel-level error analysis for the PCC (top) and CCC (bottom). A high level of agreement and correlation was observed at earlier time points (1-month) versus at later time points (5-month). MATLAB R2019b (Mathworks, Natick, MA) was used for producing individual figures, images, and graphs. Adobe Photoshop 2020 (Adobe, San Jose, CA) was used to arrange individual panels, draw schematics, and add text.

Figures 3 and 4 report the results of the model fit for scenario 1. Figure 3 shows representative model calibration results for patient 1 the best model (i.e., the most parsimonious model). The left column shows the measured total tumor cell distribution over eight slices at the baseline, 1-month, and 3-month time points. The middle column shows the model fit at 1-month and 3-months. The right column shows plots of the model determined versus measured tumor volume fractions. The model fit resulted in less than 7.9% absolute error in tumor volume in the enhancing and non-enhancing regions, resulting in Dice values of greater than 0.91 and 0.77 in the enhancing and non-enhancing regions, respectively. At the local level, a strong level of agreement and correlation was observed throughout the tumor resulting in PCCs greater than 0.88 and CCCs greater than 0.68. We note that the non-linearity observed in the scatter plots of Fig. 3 when the measured total volume fraction is equal to 0.16, is due to the model estimating non-zero enhancing disease (*ϕ*_*E*_) in regions that are indicated as non-enhancing disease in the measurement.

Figure 4 reports the error analysis for the cohort using the best model. Low global level errors were observed for the enhancing region with the median percent error in tumor volume ranging from − 1.2 to 0.14% and the median Dice values ranging from 0.78 to 0.79 across all time points. However, larger global level errors were observed for the model calibration to the non-enhancing region with median percent error in tumor volume ranging from 14.2 to 55.9% and the median Dice values ranging from 0.76 to 0.81. Low local level errors were observed with the median PCC values ranging from 0.80 to 0.86 and the median CCC values ranging from 0.58 to 0.71.

### Scenario 2 and 3: evaluation of model predictions

**Figure 5:**
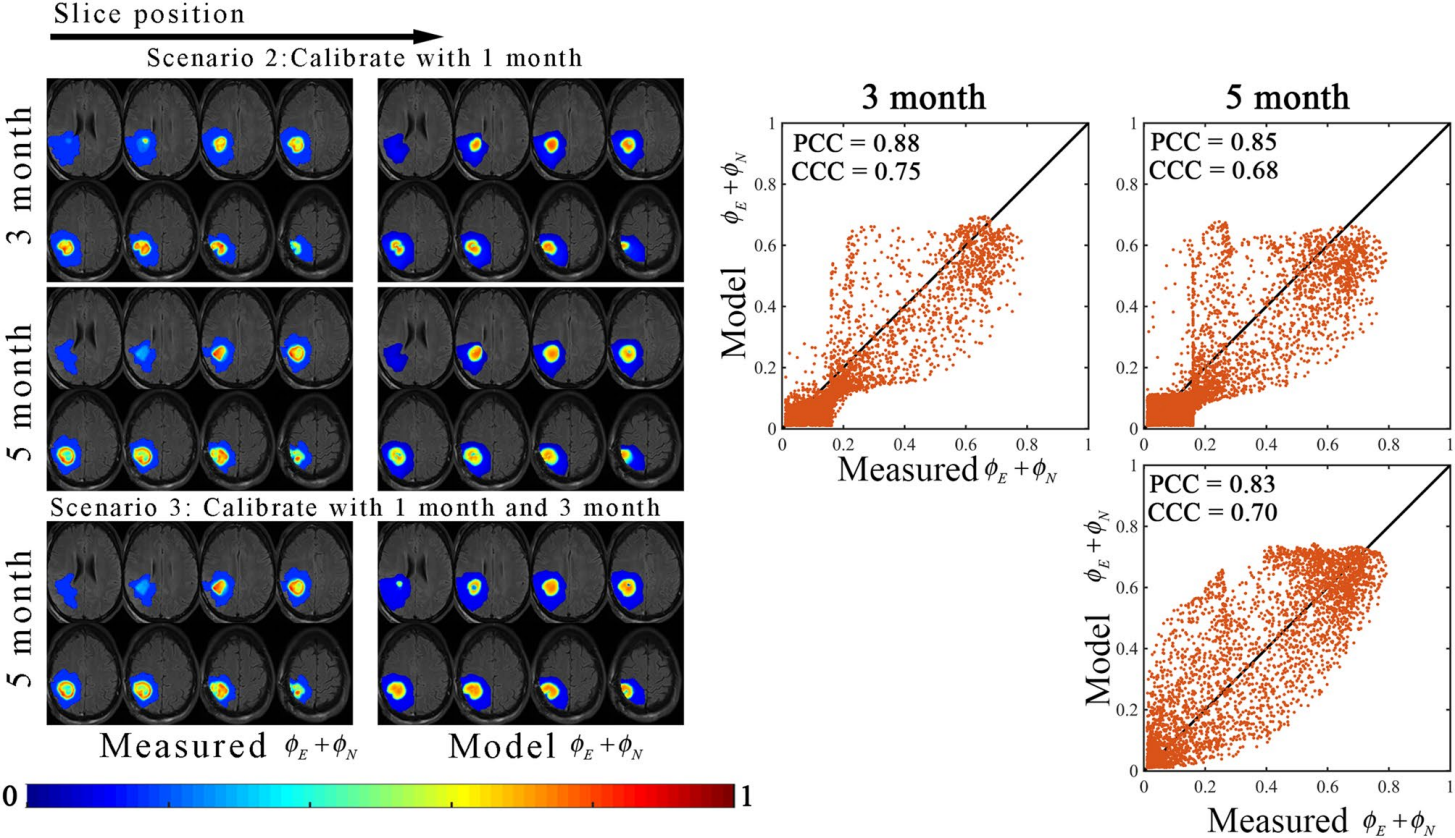
Representative results from scenarios 2 and 3. The central eight tumor slices from patient 2 are used to demonstrate the model calibration results using the best model as determined by the AIC. The measured and model determined sum of the enhancing and non-enhancing regions (i.e., *ϕ*_*E*_ and *ϕ*_*N*_) are shown in left and middle columns, whereas the voxel-wise comparison between the model determined and measured tumor distributions are shown in the right column. In general, a high level of correlation resulted in PCCs greater than 0.83 with strong agreement at the voxel level CCCs greater than 0.68. MATLAB R2019b (Mathworks, Natick, MA) was used for producing individual figures, images, and graphs. Adobe Photoshop 2020 (Adobe, San Jose, CA) was used to arrange individual panels, draw schematics, and add text.

**Figure 6:**
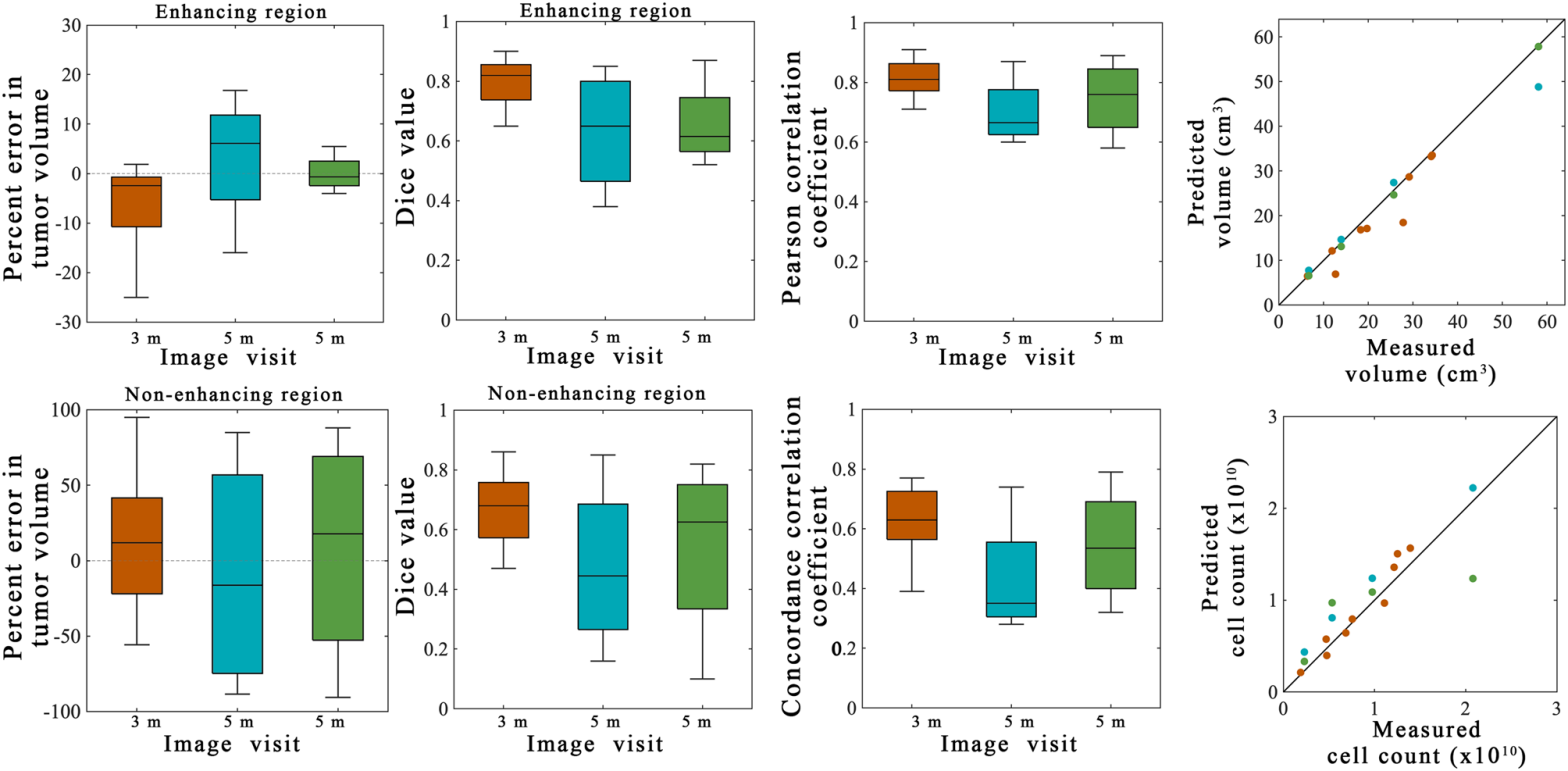
Summary statistics from scenarios 2 and 3. Error analysis from the model prediction scenarios are shown for the entire cohort. The orange bars and points represent 3-month predictions made from calibrations using the 1-month visit for calibration (scenario 2). The blue bars and points represent 5-month predictions made from calibrations using the 1-month visit for calibration (scenario 2). The green bars and points represent 5-month predictions made from calibrations using the 1-month and 3-month visits for calibration (scenario 3). The left column shows the percent error in the tumor volume for the enhancing (top) and non-enhancing (bottom) regions. Greater error was observed in the non-enhancing regions versus the enhancing regions. The second column shows the Dice values for the enhancing (top) and non-enhancing (bottom) regions. A high level of overlap (median Dice values greater than 0.62) were observed for the enhancing regions while the non-enhancing regions had greater error (Dice greater than 0.45). The third column shows the results of the voxel-level error analysis for the PCC (top) and CCC (bottom). The fourth column shows the predicted versus measured tumor volume (top) and cell count (bottom) within the enhancing region. For the 3-month predictions (orange dots) KCCs for tumor volume and cell count predictions were 0.94 and 0.92, respectively. For the 5-month predictions (blue dots) the KCCs for tumor volume and cell count predictions were 1.00 and 0.88, respectively. For the 5-month predictions (blue dots) the KCCs for tumor volume and cell count predictions were 1.00 and 0.79, respectively. MATLAB R2019b (Mathworks, Natick, MA) was used for producing individual figures, images, and graphs. Adobe Photoshop 2020 (Adobe, San Jose, CA) was used to arrange individual panels, draw schematics, and add text.

Figures 5 and 6 report the results of the model prediction for scenario 1. Figure 5 shows representative model prediction results for patient 2 based on the model with the lowest average *AIC*. The left and middle columns show the measured and predicted total tumor cell distributions, respectively, at the 3-month and 5-month time points. The right column shows plots of the predicted versus measured tumor volume fractions. For scenario 2 (i.e., when the 1-month post-treatment visit is used to calibrate the model), we observed less than 13.0% error in enhancing tumor volume. The model overestimated the non-enhancing region, resulting in 85.0% error in non-enhancing clinical tumor volume at the 5-month visit. The Dice values were greater than 0.82 at all imaging visits. We observed at the local level a high level of spatial agreement (PCCs and CCCs greater than 0.85 and 0.68, respectively), although the model fails to describe the area of necrosis at the 5-month visit. For scenario 3 (i.e., when both the 1-month and 3-month visits are used to calibrate the model), there was a − 4.0% error in enhancing tumor volume and a Dice value of 0.82 in the enhancing region. Similarly, we observed 87.9% error in non-enhancing clinical tumor volume and a Dice value of 0.80 in the non-enhancing region. A high level of correlation (PCC = 0.83) was observed, while agreement was higher (CCC = 0.70) compared to the first prediction scenario. Visualization of the remaining patients are shown in Supplemental Figures S3 to S6.

Figure 6 reports the error analysis from the prediction scenarios for the cohort using the model selected with the lowest average AIC. The median percent error in enhancing tumor volume ranged from − 2.5 to 6.1% and the median Dice values ranged from 0.62 to 0.82 for both prediction scenarios for the enhancing tumor region. We observed higher error resulting in median percent error in non-enhancing clinical tumor volume ranging from − 16.1 to 17.7% and median Dice values ranging from to 0.45 and 0.68 for the non-enhancing tumor region. At the local level, the median PCC values ranged from 0.67 to 0.81 and the median CCC values ranged from 0.45 to 0.81. The fourth column shows the predicted tumor volume and the predicted cell count versus the measured values within the enhancing region. A strong agreement and correlation were observed between the predicted and measured tumor volume (KCC ranging from 0.94 to 1.00). Similarly, a strong level of agreement and correlation was observed for tumor cell count for both the 3-month visit and the 5-month visit (when both 1-month and 3-month data were used for calibration) resulting in KCCs ranging from 0.79 to 0.92.

## Discussion

With the aim of integrating the observed inter- and intra-tumoral heterogeneity of high-grade gliomas, we have developed and systematically evaluated a computational approach that integrates commonly used multiparametric MRI data to generate a biologically-based, spatially-informative personalized model in high-grade glioma patients to assess and predict tumor response to radiation and chemotherapy at an individual patient level. A family of models with various degrees of complexity, ranging from a single-species reaction–diffusion model to a two-species reaction–diffusion model, was initialized and calibrated individually for a preliminary cohort of nine patients with high-grade gliomas using serial DWI estimates of cellularity collected in the standard-of-care setting. We then used the Akaike Information Criterion to select the model that best balanced model fit and complexity. A novel two-species model describing the enhancing and non-enhancing tumor regions was selected as the most parsimonious and was used to predict future tumor growth and response at 3-month and 5-month visits post-radiotherapy. Compared to the other coupling approaches, the selected model incorporates additional imaging information via the enhancement ratio to spatially vary the efficacy of RT and CT. At the 3-month prediction, we observed a median error of − 2.5% (interquartile range, IQR, of 10.0%) in tumor volume predictions and a median PCC of 0.81 (IQR of 0.10) was observed for voxel-level predictions in the enhancing region. At the 5-month prediction for scenario 3, we observed a median error of − 0.7% (interquartile range, IQR, of 4.9%) in tumor volume predictions and a median PCC of 0.76 (IQR of 0.20) was observed for voxel-level predictions in the enhancing region.

Image-based mathematical models of high-grade glioma growth^20^ have resulted in several promising insights into response to radiotherapy^16,24,56^, mass effect^11,28^, angiogenesis^26,27^, and treatment efficacy^57^. At the clinical level, a majority of these approaches employ methods only sensitive to the presumed extent of tumor burden (i.e., contrast enhanced *T*_*1*_-weighted or *T*_*2*_-FLAIR MRI) and not any quantitative imaging measures that are sensitive to local tissue composition. We hypothesize that knowledge of the spatial and temporal dynamics of the intratumoral heterogeneity and the associated mechanistic relationships may be particularly important in the development of localized therapeutic approaches. Indeed, it is well known that the efficacy of standard-of-care therapies may vary spatially due to hypoxia^58^ or distribution of chemotherapeutic agents^59^ leading to disease progression. Spatially-mapping regions of forecasted tumor resistance to the current therapy could be used to target treatment intensification with optimal sequencing of novel systemic therapy in combination with local treatment intensification using focal radiotherapy approaches^4,5^ or laser interstitial thermal therapy^60^. To that end, we leveraged standard-of-care DWI data (in addition to measures of tumor extent) to provide estimates of cell density within the tumor and therefore intratumoral heterogeneity. With this approach we observed high accuracy in model calibration (Figs. 3 and 4) and prediction (Figs. 5 and 6) at the global and voxel levels.

An additional novel aspect of this work is the development of a two-species model of tumor growth and response that characterizes the change in the enhancing and non-enhancing regions. While there are other approaches that characterize multiple species (e.g., hypoxic, normoxic and vasculature^26^; proliferative, necrotic, edema^61^; proliferative/invasive or go-or-grow^62^; tumor and vasculature^27^), we arrived at modeling these two distinct imaging regions based on the available data from standard-of-care imaging and clinical considerations in tumor response assessment. Although, if additional imaging data characterizing tumor vasculature^63^ or hypoxia^8,64^ were accessible, these models may facilitate improved predictions of regions of necrosis or hypoxia that are currently not explicitly captured by our two-species model. We note that in our pre-clinical efforts we have incorporated dynamic contrast-enhanced MRI to characterize tumor vasculature and have observed an improvement in tumor growth and response predictions^16,27^. A similar approach to our two-species model would be a “go-or-grow” model (such as^62^), where the enhancing region could be considered the “grow” phenotype and the non-enhancing region the “go” phenotype. Some of the key differences between the model presented in this manuscript and the go-or-grow model is that we do not assume that the two regions only proliferate or migrate, and we do not assume cells transition between species. One limitation to implementing a go-or-grow model is that additional rules and (potential) assumptions on parameter fields are needed to define how the cells transition between phenotypes in response to, for example, cell density^65^ or a nutrient field^62^. We note, that our single species model with a global proliferation rate and uniform effects of RT and CT is comparable to the reaction–diffusion (or proliferation-invasion) models used by others^10,24^, with the exception of using DW-MRI to assign tumor cell density. This model, however, was not ranked in the top ten of models (Supplemental Table S4).

There are several opportunities to improving on this experimental-computational approach. First, like most imaging measures, DWI is sensitive to a range of complex phenomena. As we have previously discussed in detail elsewhere^27^, we assume the predominant influence on the *ADC* is cell density. But changes in the *ADC* may also occur due to changes in cell size, cell permeability or tortuosity of the tissue, as well as nearby structural elements such as hemorrhage, should be acknowledged^27^. Second, our models of response to radiotherapy and chemotherapy are likely an oversimplification of tumor response. For example, neither the synergistic effects of temozolomide on radiotherapy are considered^66^, nor are the temporal dynamics of response to radiation therapy (e.g., repair, reoxygenation)^67^. Third, there may be an incomplete description of the high-grade glioma biology that should be investigated in future iterations. This includes (for example) the reduction of the tumor to enhancing and non-enhancing disease, exclusion of patients with resected disease, exclusion of angiogenesis^26,27^, disease subtype^68^, and patient sex^69^. We posit that some factors such as disease subtype and patient sex may be captured implicitly in the calibrated model parameters; however, a larger cohort would be needed to effectively assess that hypothesis. Additional imaging data (such as perfusion imaging^63^) may also be needed to incorporate patient-specific models of angiogenesis^23^ or hypoxia^8,64^. However, the availability of the required data types should be considered when increasing model complexity. Based off of our previous pre-clinical studies^16,27^, we hypothesize that spatially and temporally evolving the proliferation rates in response to vascular dynamics would improve the voxel level agreement of tumor predictions (as observed in Figs. 3 and 5). Fourth, while we attempted to employ semi-automated approaches (further details in Supplementary Materials S1), the use of manual imaging segmentations contributes to uncertainty in the measured data and more repeatable approaches should be considered. The development of semi-automated or automated approaches is an area of active research in the field (e.g., the Brain tumor image segmentation (BRATS) challenges^70^), including co-authors of this manuscript, which could improve both workflow efficiency and repeatability of segmentations. Finally, model weighting approaches that utilize the AIC (or other model selection metrics) should be considered to generate ensemble forecasts. In this manuscript, the first and second rank model had very similar AIC values (Supplemental Table S4), which suggest they might both be valid models of response. Model weighting would allow the generation of an ensemble forecast that takes in account both of these models.

## Conclusions

We have developed and evaluated a biologically-based, spatially-informative mathematical model of tumor growth and response to chemotherapy and radiation therapy that can be parameterized for individual patients via standard-of-care anatomical and quantitative MRI data. As a proof of concept, we applied this novel computational and imaging-based pipeline in nine high-grade glioma patients. This patient-specific approach achieved a low median error in tumor volume forecasts of less than 2.5% using a two-species model of tumor growth. This work demonstrates the plausibility of using clinically accessible MRI data to initialize and constrain predictive mathematical models of tumor growth and response in high-grade gliomas. A prospective study in a larger cohort is needed to validate this predictive modeling framework.

## Supplementary Information

Supplementary information.

## Data Availability

The datasets generated during and/or analyzed during the current study are available from the corresponding author on reasonable request.
