# Supplementary material for: Image-based personalization of computational models for predicting response of high-grade glioma to chemoradiation
Source: Sci Rep. 2021 Apr 19;11:8520. doi: 10.1038/s41598-021-87887-4 (PMC8055874; doi:10.1038/s41598-021-87887-4)
Supplement: Supplementary file 1 — Supplementary information. [file 41598_2021_87887_MOESM1_ESM.pdf]

Supplemental material: Image-based personalization of computational models for predicting response of high-grade glioma to chemoradiation

David A. Hormuth II[1,5], Karine A. Al Feghali[7], Andrew M. Elliott[7],
Thomas E. Yankeelov[1-6α], Caroline Chung[7α],
[1]Oden Institute for Computational Engineering and Sciences
Departments of [2]Biomedical Engineering, [3]Diagnostic Medicine, and [4]Oncology
[5]Livestrong Cancer Institutes
The University of Texas at Austin, Austin, Texas
Departments of [6]Imaging Physics and [7]Radiation Oncology
MD Anderson Cancer Center, Houston, Texas
[α]Co-senior authors

* Please address correspondence to:
David A. Hormuth, II, Ph.D
The University of Texas at Austin
201 E. 24th Street
POB 4.102
1 University Station (C0200)
Austin, Texas 78712-1229
e-mail: david.hormuth@utexas.edu

S.1. MRI data and processing

MRI data was acquired on eight different MRI scanners: 1) a 1.5T GE Optima MR450w, 2) a 1.5T GE Signa Excite, 3) a 1.5T Signa Excite HDxt, 4). A 3T GE Discovery MR750, 5) a 1.5T Siemens Aera, 6) a 1.5T Siemens Espree, 7) a 3T Siemens Prisma, and 8) a 3T Siemens TrioTim. We used four MRI data types in our analysis that were acquired at each scan session: 1) a pre-contrast T_1-weighted image, 2) a post-contrast T_1-weighted image, 3) T_2-FLAIR, 4) DWI. In this retrospective cohort, the scan settings (e.g., *TR, TE*, acquisition matrix) for each sequence were not identical across patients, therefore the individual scan settings for each sequence is not reported. However, the images were all acquired based on standard-of-care, diagnostic quality, images used at the time of acquisition. A Gadolinium based contrast agent (Gadovist) was used for the contrast-enhanced, T_1-weighted image series. A rigid

registration algorithm (*imregister* in MATLAB (Mathworks, Natick, MA)) was used to provide intra- and inter-visit registration. For intra-visit registration, each image and parameter map were registered to the T_2-FLAIR image collected at that visit. The intra-visit registration consisted of resampling of images to match the image resolution of the T_2-FLAIR image. For the inter-visit registration, each image and parameter map acquired at post-baseline image sessions were registered to the T_2-FLAIR image acquired at baseline. Panel A in Figure 1 shows an example of the registration process for a given patient.

For each patient, the gross tumor volume (GTV) was defined as the enhancing tumor on the post-contrast T_1-weighted images and clinical tumor volume (CTV) defined as non-enhancing, T_2-hyperintense region on the T_2-FLAIR images were segmented using a semi-automated approach using thresholding methods in combination with manual adjustments by a radiation oncologist and secondary quality review by a second senior radiation oncologist. A *k*-means clustering of signal intensity was used to segment the white matter, gray matter, and cerebrospinal fluid from T_2-FLAIR images [1]. The brain-skull interface was manually segmented from the T_2-FLAIR image. Panel B in Figure 1 shows an example of this segmentation process.

The *ADC* calculated from DWI data was used to estimate the tumor cell volume fraction at each imaging visit. *ADC* was estimated voxel-wise from an echo planar imaging based DWI sequence using Eq. (1):

$$ADC = \frac{\ln\left(S_{b_1}/S_{b_2}\right)}{b_2 - b_1}, \tag{1}$$

where b_1 and b_2 are the *b*-values of 0 and 1000 s/mm^2, respectively, and S_{b_1} and S_{b_2} are the signal intensities corresponding to b_1 and b_2, respectively. The tumor volume fraction at 3D position $\bar{x}$ and time t, $\phi_T\left(\bar{x},t\right)$, was then estimated on a voxel-specific basis as described in [2–5], using the *ADC* of free water (ADC_w) [6], the minimum ADC measured (ADC_{min}), and Eq. (2):

$$\phi_T(\bar{x},t) = \left(\frac{ADC_w - ADC(\bar{x},t)}{ADC_w - ADC_{\min}} \right) . \quad (2)$$

We have used this approximation previously to provide non-invasive estimates of tumor cellularity [3–5,7,8]; however, we note that this approximation is a simplification of all the biological aspects that contribute to changes in *ADC*. This point is discussed further in [3,9]. Within the GTV, we assumed that the primary cellular contribution is from tumor cells, therefore $\phi_T(\bar{x},t)$ was calculated using Eq. **(2)**. However, within the CTV the cell density or relationship to imaging features is less clear, thus we used a fixed value of 0.16 [10] everywhere within that region. An alternative approach for assigning celluarity in the non-enhancing regions (used in [10,11] and elsewhere) is to assume a spatially varying value of cellularity decreasing from the value observed at the interface of the enhancing region to a fixed value at the periphery of the non-enhancing region. For the two-species model, the tumor volume fraction within the enhancing or GTV region was calculated using Eq. (2) and set to zero outside the GTV, while in the non-enhancing or CTV region, it was set to a fixed value of 0.16.

S.2. Numerical implementation

The spatial-temporal evolution of $\phi_T(\bar{x},t)$ was determined using a 3D finite difference approximation implemented in MATLAB R2019b (Mathworks, Natick, MA) using a fully explicit in time differentiation and central difference spatial differentiation. The numerical time step was selected to maintain numerical stability based on the grid spacing and diffusion coefficient values. No flux (Neumann) boundary conditions were used for $\phi_T(\bar{x},t)$ at the skull boundary. The boundary condition for $\vec{u}$ was assumed to be zero displacement in the normal direction, while it was assumed that the tissue in the tangential directions was free to move (i.e., slip condition). Identical numerical approaches were also used for $\phi_E(\bar{x},t)$ and $\phi_N(\bar{x},t)$. For a complete description of the numerical implementation of these techinques, the interested reader is referred to [12].

Supplemental Table 1: Model combinations

Model number	Coupling approach	Model number	Coupling approach
1	$C_{RT} = C_1$ $C_{CT} = C_4$	6	$C_{RT} = C_4$ $C_{CT} = C_3$
2	$C_{RT} = C_2$ $C_{CT} = C_4$	7	$C_{RT} = C_1$ $C_{CT} = C_1$
3	$C_{RT} = C_3$ $C_{CT} = C_4$	8	$C_{RT} = C_2$ $C_{CT} = C_2$
4	$C_{RT} = C_4$ $C_{CT} = C_1$	9	$C_{RT} = C_3$ $C_{CT} = C_3$
5	$C_{RT} = C_4$ $C_{CT} = C_2$	10	$C_{RT} = C_4$ $C_{CT} = C_4$

S.3 Model parameter calibration

A total of 40 models (Figure 1C) were developed from two base models (i.e., one-species or two-species), 10 therapy coupling combinations (i.e., C_1 to C_4), and two proliferation parameterization approaches (described below). First, we assumed $k_{p,T}$ and $k_{p,E}$ were uniform throughout the domain. Second, we assumed that $k_{p,T}$ and $k_{p,E}$ varied as a field within the tumor regions of interest. When the proliferation rates were calibrated as a field, we only calibrated for a subset of points within the tumor and then interpolated elsewhere. For example, for a given 3 × 3 voxel sub-region within the tumor, the parameter values were calibrated at the corner and center positions while the remaining four points were interpolated from the nearest calibrated values [9]. This parameter calibration approach significantly reduces the number of individual parameters and results in a spatially smooth parameter field. To reduce the number of model parameters for the two-species model, we assumed that the $D_{N,w}$ and $D_{N,g}$ are proportional to $D_{E,w}$ and $D_{E,g}$, respectively, by a single calibrated scalar factor. Likewise, we assumed $k_{p,N}$ is proportional to $k_{p,E}$. β_{NE} and β_{EN} were empirically determined and assigned to values of 4 and 1, respectively. The remaining calibrated model parameters (in Table 2) were fit as a global variable. Model parameters are bounded as shown in Supplemental Table 2 based on their physical definition, numerical stability, or within an order of magnitude of values reported in literature.

Supplemental Table 2: Model parameter bounds for calibration

Model parameter	Parameter bounds [Lower Upper]
$k_{p,T},\ k_{p,E}\ \ k_{p,N}$ (day^{-1})	[0 10]
$\theta_{T},\ \theta_{E},\ \theta_{N}$	[0 1]
$D_{T,w}, D_{T,g}, D_{E,w}, D_{E,g}, D_{N,w}, D_{N,g}$ (mm^2/day)	[0 $D_{stability}$]
λ_1	[0 10]
$SF_{RT,min}$	[0 1]
$SF_{CT,min}$	[0 1]

The upper bound for the diffusion coefficients was assigned as $D_{stability}$. $D_{stability}$ is the maximum allowable diffusion coefficient to maintain numerical stability and must satisfy the condition below:

$$0.25 > D_{stability}\Delta t\left(\frac{1}{\Delta x^2}+\frac{1}{\Delta y^2}+\frac{1}{\Delta z^2}\right), \tag{3}$$

where Δt is the simulation time step, Δx is the image resolution in the x-direction, Δy is the image resolution in the y-direction, and Δz is the image resolution in the z-direction.

Panel D in Figure 1 depicts the calibration approach used in this study. For each patient, we considered three different calibration/prediction scenarios. For the first scenario, we calibrated each model to all of the available data to see how well the models describe that data. For the second and third scenarios, we calibrated each model to a subset of the available data (baseline and 1-month for scenario 2; baseline, 1-month, and 3-month for scenario 3) and then those calibrated parameters are used to run the model forward in time to predict the tumor response at that patient's remaining imaging visits (i.e., the 3-month and 5-month visits).

While the salient details of the calibration approach are highlighted here, a more complete description of the calibration algorithm can be found in the referenced publication [12]. Figure 2 provides a schematic of our model calibration approach. Briefly, we used the Levenberg-Marquardt [12,13]

algorithm to minimize the sum of the squared errors between the measured and simulated tumor growth (i.e., $\phi_T(\bar{x},t)$ in Eq. (2), or $\phi_E(\bar{x},t)$ and $\phi_N(\bar{x},t)$ in Eqs. (5) – (6))). First, an initial guess of model parameters and the initial conditions at baseline are used in a finite difference simulation of either the single or two-species model above. Second, the finite difference simulation of tumor growth is sampled at the imaging visits used for model calibration (i.e., 1-month and 3-month visits for scenario 1 for this patient). Third, model error is assessed and used within the Levenberg-Marquardt algorithm to update model parameters to minimize the sum squared error. The algorithm ceases when either the error in the objective function stagnates (less than 0.5% change in successive iterations) or when 1000 iterations are reached. For the second and third calibration scenarios, the calibrated parameters were then used to run the model forward in time to predict tumor growth at the remaining time points not used for model calibration. For the single species model our objective function is defined as:

$$Error = \sum_{t_i}^{t_f} \sum_{x=1}^{n} \left(\left(\phi_T(\bar{x},t,\beta) - \phi_{T,meas}(\bar{x},t) \right)^2 \right), \quad (4)$$

where $\phi_T(\bar{x},t,\beta)$ is the model estimated tumor volume fraction for a given set of parameters β at 3D position $\bar{x}$ and time t, t_i is the index for the first time point used in the calibration, t_f is the index for the final time point used in the calibration, and $\phi_{T,meas}(\bar{x},t)$ is the measured tumor volume fraction from DW-MRI. For the two species model, the objective function is defined as the summation of the error in the enhancing region and the non-enhancing region as shown below:

$$Error = \sum_{t_i}^{t_f} \sum_{x=1}^{n} \left(\left(\frac{\phi_E(\bar{x},t,\beta) - \phi_{E,meas}(\bar{x},t)}{\theta_E} \right)^2 + \left(\frac{\phi_N(\bar{x},t,\beta) - \phi_{N,meas}(\bar{x},t)}{\theta_N} \right)^2 \right), \quad (5)$$

where $\phi_E\left(\overline{x},t,\beta\right)$ and $\phi_N\left(\overline{x},t,\beta\right)$ are the model estimated tumor volume fractions for a given set of parameters β for the enhancing and non-enhancing region, respectively, and $\phi_{E,meas}\left(\overline{x},t\right)$ and $\phi_{N,meas}\left(\overline{x},t\right)$ are the measured tumor volume fractions for the enhancing and non-enhancing regions, respectively. We note that in Eq. (5) the residuals between both species are normalized by their carrying capacity to account for magnitude differences in the two volume fractions.

S.4 Sensitivity of model calibration to measurement noise

We evaluated the model calibration approach using an *in silico* tumor (i.e., a tumor whose growth is governed by the models above with user-selected parameter values). The purpose of this *in silico* study was to evaluate the ability of our parameter calibration approach to accurately determine the true model parameter governing the *in silico* tumor's growth and response. For this *in silico* study we assessed the robustness of the parameter calibration in the presence of 10% measurement noise. For the model with the most parameters (*i.e.,* the two-species model with a calibrated proliferation field), 250 noisy datasets were generated by adding random noise from a Normal distribution with a standard deviation of 10%. (In our study, the largest variation we observed for a given patient in voxel-wise values of ADC for normal appearing white matter was a mean difference of -2.9% with a standard deviation of 9.7% compared to the baseline image. Thus, the 10% value is appropriate.) Model parameters were then estimated from the noisy datasets and then compared to the known parameters used to "grow" the *in silico* tumor. Initial guesses for model parameters for each calibration scenario were selected randomly from a uniform distribution with lower and upper bounds shown in Supplemental Table 2 to ensure parameter guess were not near the true values. We observed that less than 5.6% error was observed when two time points were used for calibration or less than 3.33% when three or more time points were used for calibration. Supplemental Table 3 reports the full results for this exercise.

Supplemental Table 3: *In silico* parameter estimation error

	Calibration Scenario		
Parameter	1-month	1-month & 3-month	1-month, 3-month, & 5-month
$k_{p,E}$	2.17 ± 0.42	0.21 ± 0.37	0.07 ± 0.36
$D_{E,W}$	-0.16 ± 6.46	-2.33 ± 2.16	-1.17 ± 0.71
$D_{E,G}$	-3.36 ± 2.23	-3.33 ± 3.24	-1.78 ± 0.96
$k_{p,N}$	-5.60 ± 1.01	-1.31 ± 0.36	1.05 ± 0.82
D_N	-0.58 ± 1.39	-0.18 ± 0.75	-0.06 ± 2.57
λ_1	4.54 ± 3.15	-0.49 ± 1.03	0.53 ± 0.97
θ_E	2.55 ± 3.11	1.01 ± 0.98	1.19 ± 0.23
SF_{RT}	0.52 ± 0.21	0.12 ± 0.06	-0.10 ± 0.13
SF_{RT}	0.04 ± 0.09	0 ± 0.98	-0.68 ± 0.05

Mean ± 95 confidence interval

This *in silico* study indicates that for a range of noise expected from standard-of-care MRI data, we are able to calibrate for voxel-wise parameters and accurately estimate treatment effects for individual patients.

S.5 Model selection

The Akaike Information criterion (*AIC*, [14]) was used to select the model that optimally balances model complexity and model-data agreement. The *AIC* was calculated using:

$$AIC = 2k + n\ln\left(\frac{RSS}{n}\right) + 2k\left(\frac{k+1}{n-k-1}\right), \tag{6}$$

where k is the number of parameters calibrated for a given model, n is the number of data points used to calibrate the model, and RSS is the residual sum squares between the measured and model estimated tumor growth. We calculated the *AIC* for each model over the timepoints used for model calibration. We then selected the model with the lowest average *AIC* across all patients as the most parsimonious model. We then calculated the Akaike weights for each model defined as:

$$w_i = \frac{\exp\left(-\frac{\delta_i}{2}\right)}{\sum_{j=1}^{40}\exp\left(-\frac{\delta_j}{2}\right)} , \tag{7}$$

where w_i is the weight for the *i-th* model, δ_i is equal to AIC_i - AIC_{min}, and $AIC_{\min}$ is the minimum observed AIC. The model with the lowest average *AIC* was the two species model, with a locally varying proliferation rate and radiation and chemotherapy both coupled to approach C2 (i.e., coupled to *ER*). This model will be used in all of the model calibrations and predictions reported in the results. Supplemental Table 4 reports the ten model combinations with the lowest AIC. Of these ten models, six were the two-species model, four were the single-species model, and seven had a spatially varying proliferation rate.

Supplemental Table 4: Model selection results

Model rank	1	2	3	4	5
Model	2s/field/8	2s/field/1	1s/field/6	2s/field/2	1s/global/5
Average AIC	-35.56	-35.30	-23.38	-22.75	-22.57
Weight	0.54	0.45	0.0012	0.0009	0.0008
**Model rank**	**6**	**7**	**8**	**9**	**10**
Model	2s/global/5	1s/field/2	2s/field/6	2s/global/1	1s/field/8
Average AIC	-22.34	-22.07	-20.91	-19.39	-18.84
Weight	0.0007	0.0006	0.0003	0.0002	0.0001

Single species (1s) or two species (2s) model / field or global proliferation rate / coupling combination

S.6 Individual patient results for the prediction phase

Supplemental Figure 1 reports prediction results for the selected model to an assumed "no-growth" or static model. That is, for the no-growth model we assume that the tumor growth at the 3-month and 5-month visits are assumed to be identical to the 1-month visit tumor. At the three-month visits there are some patients who experience very little change in tumor volume compared to baseline (left panel in Supplemental Figure 1); however, it is clear that a no-growth model would be insufficient for 5-month predictions. The median percent error for no-growth predictions exceeds that of the model-based predictions (right panel in Supplemental Figure 1).

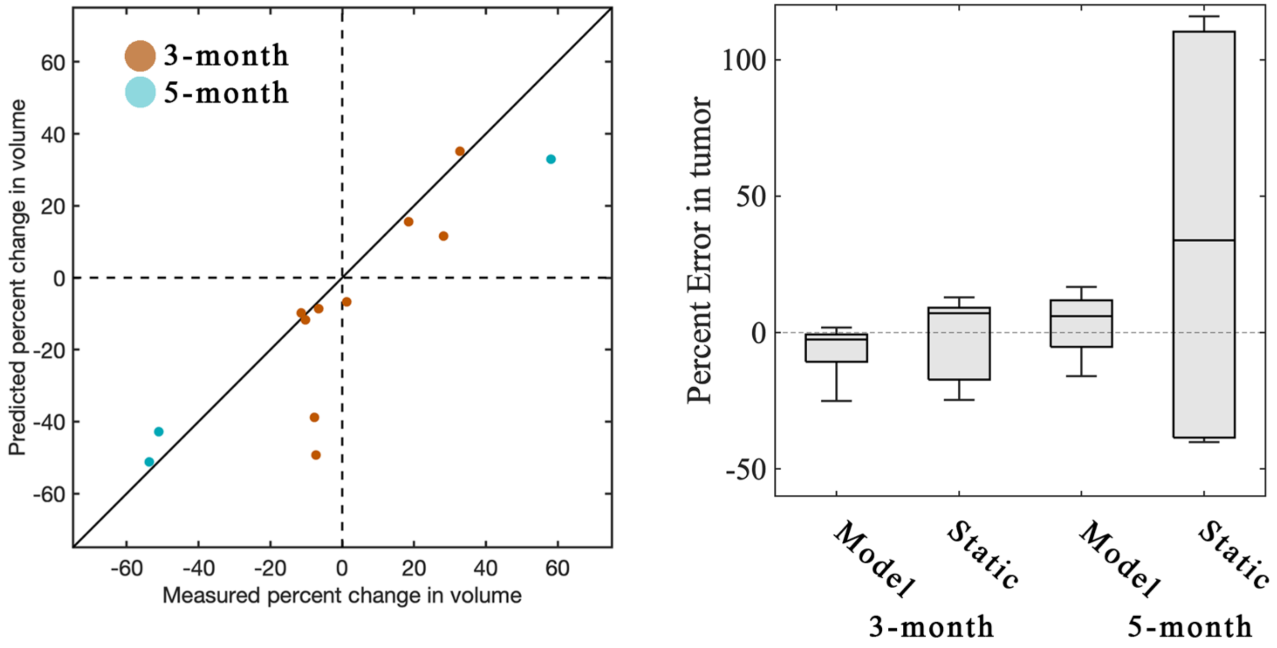

Supplemental Figure 1: Comparison of tumor growth predictions to a "no-grow" or static model. The above figures compare tumor growth predictions from the selected model to a "no-grow" or static model of response. The static or "no-grow" model assumes that the tumor does not change from the final time point used for calibration (1-month) to the prediction time points (3-month and 5-month). The left panel shows the predict percent change in volume from the 1-month time point to the 3-month (orange) and 5-month (blue) versus the measured percent change in volume. While there are a few patients near 0% change in volume from 1-month to 3-month, there are more patients with either large increases or decreases in tumor volume. The right panel, shows the percent error in tumor volume predictions the selected model versus the static or no grow model. At the 3-month and 5-month predictions, the static model has a higher median error and interquartile range.

Supplemental Figure 2 reports the effects of domain discretization on Dice similarity coefficients. A coarseness factor of 1 corresponds to the acquired image resolution while a coarseness factor of 4 corresponds to a down-sampling of the image domain by a factor of 4. Increasing coarseness does not significantly change the Dice values for either the 3-month or 5-month predictions.

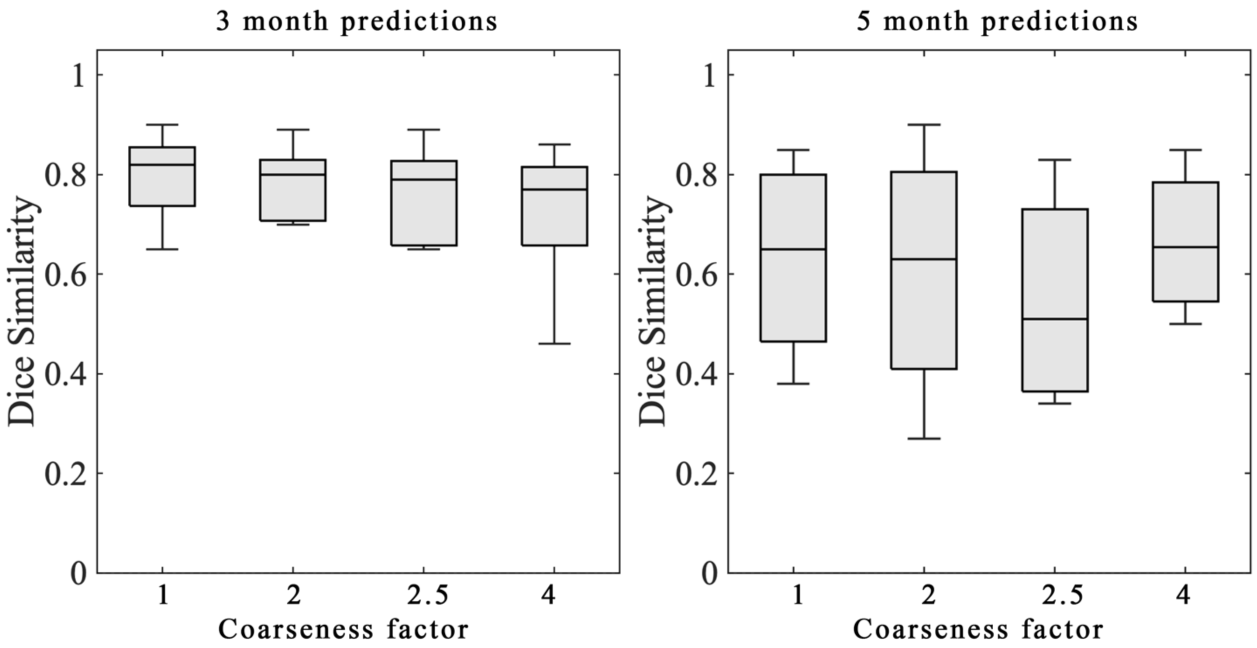

Supplemental Figure 2: Effect of discretization on Dice similarity coefficients. The above figure compares tumor growth predictions from four different discretization approaches. The coarseness factor is the is the factor by which the in-plane resolution is down sampled relative to the acquired image resolution. In the manuscript, we report results for a coarseness factor of 1 so that model estimates are on an identical grid to the image measurements. The above panels shows the prediction results when the model is calibrated to the 1-month visit and used to predict the 3-month (left panel) and 5-month (right panel) visits. For the 3-month predictions, the median Dice values range from 0.77 to 0.82, while for the 5-month predictions the median Dice values range from 0.51 to 0.66. For 3-month predictions, varying the coarseness does not appear to significantly change the results. However, a larger range of Dice values are observed for the 5-month predictions.

Supplemental Figures 3-6 report the predictions for all of the patients not reported within the manuscript. The calibrated model parameters for each patient for the selected model are reported in Supplemental Table 5.

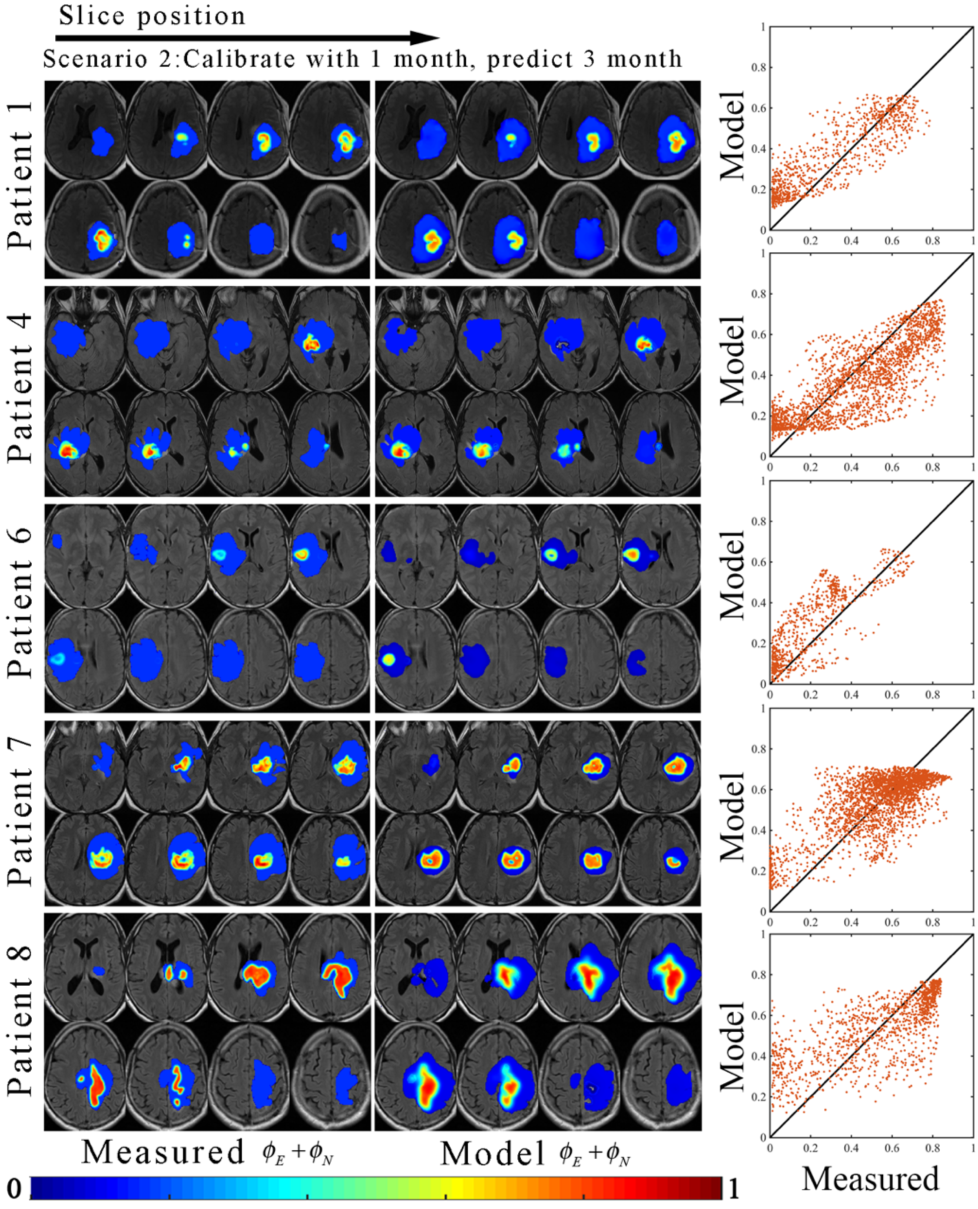

Supplemental Figure 3: Tumor growth predictions for second calibration scenario. Model parameters calibrated from the 1-month imaging visit were used to predict tumor growth at the 3-month image visit. The eight central tumor slices are shown for patients 1, 4, 6, 7, and 8. The left column shows the measured tumor growth, while the middle column shows the model estimated tumor growth. The right column shows scatter plots of the tumor volume fraction at each imaging voxel. The black line represents the line of unity.

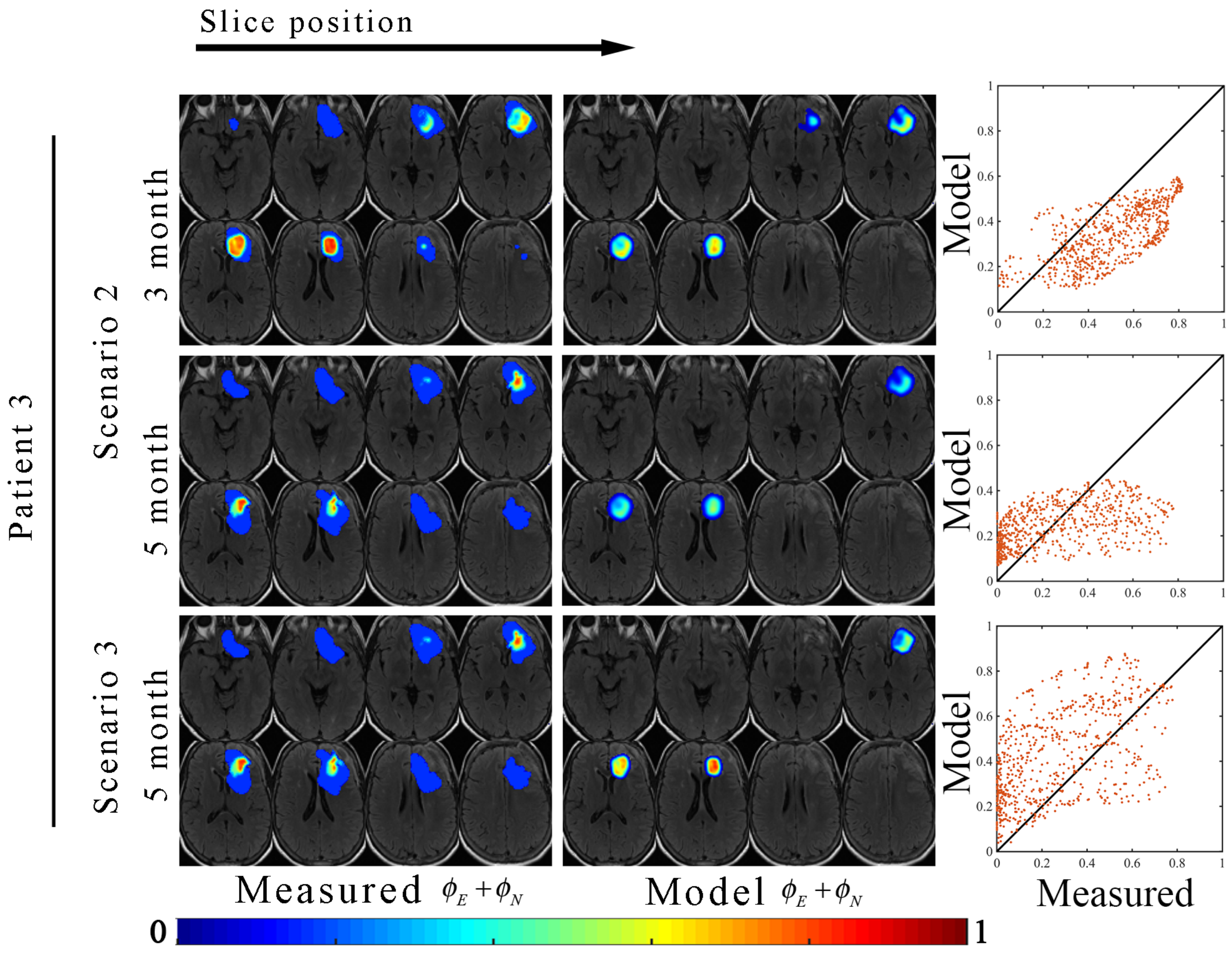

Supplemental Figure 4: Tumor growth predictions for second and third calibration scenario. Tumor growth predictions for patient 3 when calibrated to the 1-month image visit (first and second row) and when calibrated to the 1-month and 3-month image visit (third row). For each prediction, the eight central tumor slices are shown. The left column shows the measured tumor growth, while the middle column shows the model estimated tumor growth. The right column shows scatter plots of the tumor volume fraction at each imaging voxel. The black line represents the line of unity.

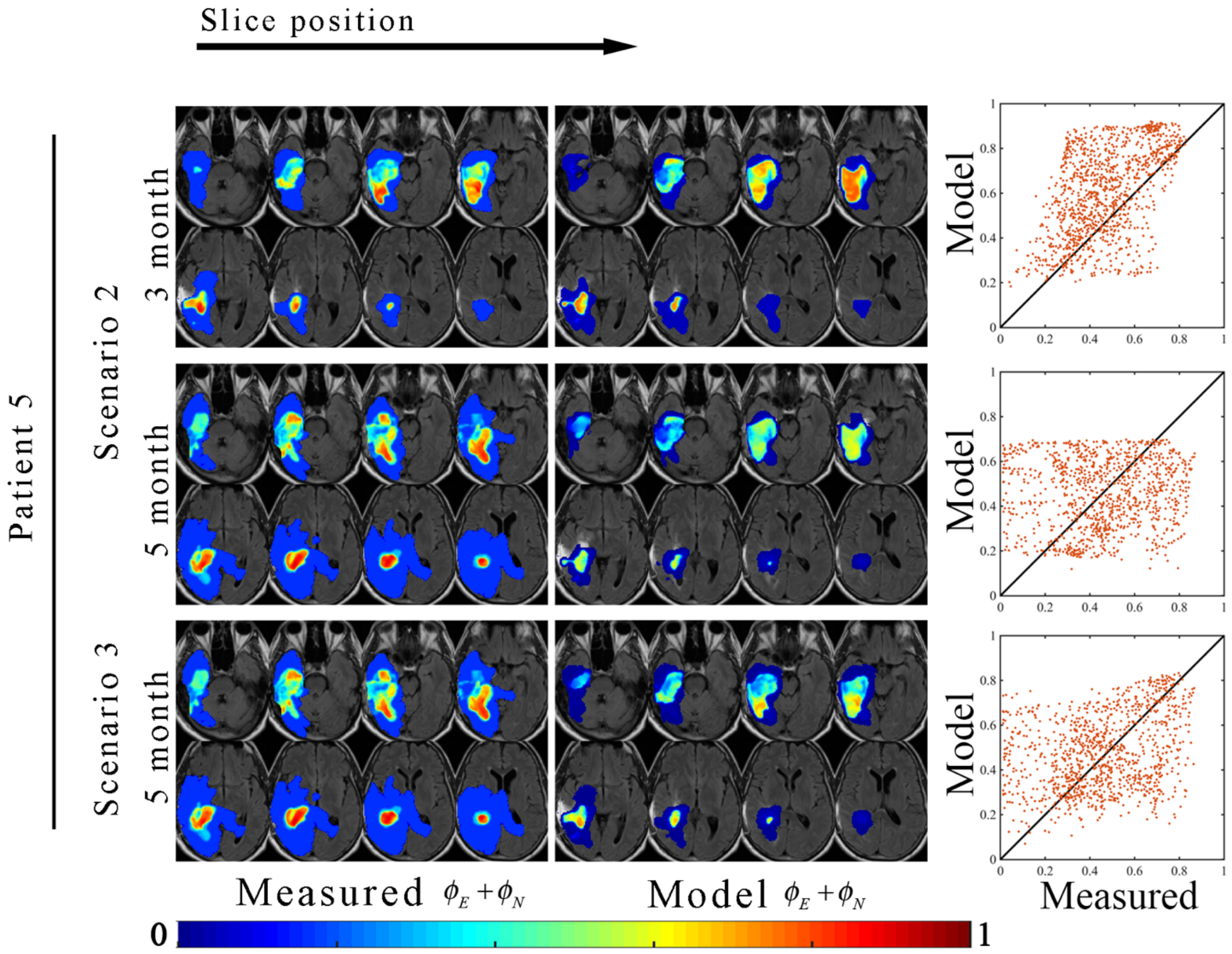

Supplemental Figure 5: Tumor growth predictions for second and third calibration scenario. Tumor growth predictions for patient 5 when calibrated to the 1-month image visit (first and second row) and when calibrated to the 1-month and 3-month image visit (third row). For each prediction, the eight central tumor slices are shown. The left column shows the measured tumor growth, while the middle column shows the model estimated tumor growth. The right column shows scatter plots of the tumor volume fraction at each imaging voxel. The black line represents the line of unity.

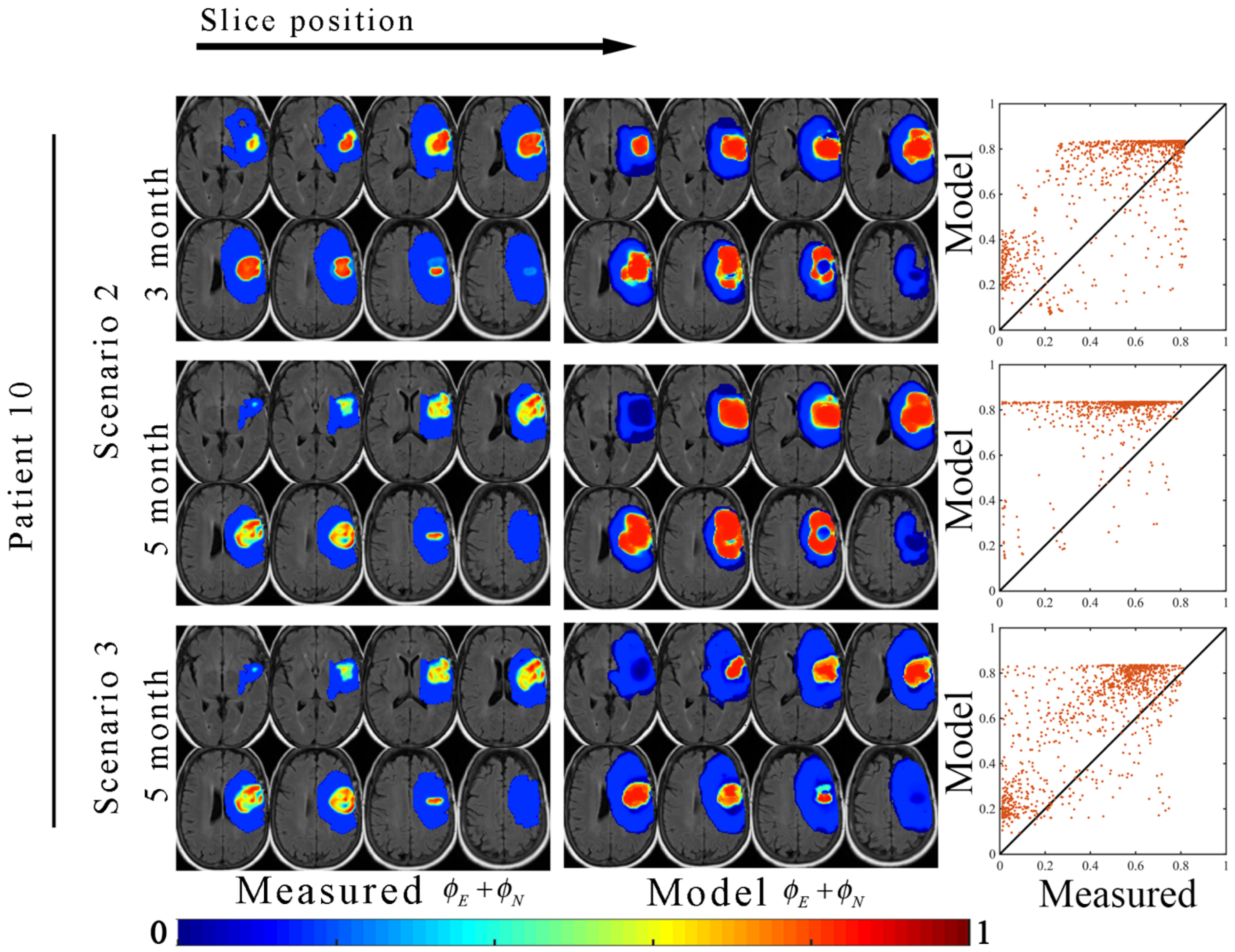

Supplemental Figure 6: Tumor growth predictions for second and third calibration scenario. Tumor growth predictions for patient 10 when calibrated to the 1-month image visit (first and second row) and when calibrated to the 1-month and 3-month image visit (third row). For each prediction, the eight central tumor slices are shown. The left column shows the measured tumor growth, while the middle column shows the model estimated tumor growth. The right column shows scatter plots of the tumor volume fraction at each imaging voxel. The black line represents the line of unity.

Supplemental Table 5: Calibrated model parameters for each patient

	Patients								
	1	2	3	4	5	6	7	8	9
$k_{p,E}$ (day^{-1})	0.91	0.83	0.76	0.95	0.96	0.50	0.89	0.49	1.42
$k_{p,N}$ (day^{-1})	2.98	3.45	1.49	2.13	1.56	0.76	1.49	1.18	1.66
θ_E	0.80	0.82	0.82	0.87	0.85	0.82	0.85	0.84	0.85
$D_{E,w}$ (mm^2/day)	0.02	0.01	0.13	0.01	0.07	0.03	0.02	0.18	0.05
$D_{E,g}$ (mm^2/day)	0.02	0.01	0.45	0.01	0.05	0.03	0.04	0.34	0.04
$D_{N,w}$ (mm^2/day)	0.29	0.31	0.12	0.12	0.28	0.29	0.06	0.18	0.29
$D_{N,g}$ (mm^2/day)	0.28	0.03	0.44	0.07	0.19	0.29	0.15	0.33	0.21
λ_1	1.24	4.00	3.24	2.21	2.01	1.90	1.12	3.45	0.50
$SF_{RT,min}$	0.9	0.99	0.97	0.99	0.93	0.96	0.98	0.99	0.99
$SF_{CT,min}$	0.74	0.79	0.91	0.86	0.71	0.84	0.94	0.60	0.60

These are the parameter values when the selected model is calibrated to the entire tumor growth time course. The model that was selected via the Akaike Information Criterion was the two species reaction-diffusion model, with a field proliferation rate, and coupling combination 8. For the two-species model the following parameters are not calibrated: $k_{p,T}$,, θ_T, $D_{T,w}$, $D_{T,g}$.. *$k_{p,E}$ is reported as an average of the parameters over that field.

References

[1] Liu J, Guo L. 2015. An Improved K-means Algorithm for Brain MRI Image Segmentation. 3rd International Conference on Mechatronics, Robotics and Automation.

[2] Hormuth II DA, Weis JA, Barnes SL, Miga MI, Rericha EC, Quaranta V, Yankeelov TE. 2015; Predicting in vivo glioma growth with the reaction diffusion equation constrained by quantitative magnetic resonance imaging data. *Phys Biol*. **12** 4 46006.

[3] Hormuth DA, Weis JA, Barnes S, Miga MI, Quaranta V, Yankeelov TE. 2018; Biophysical Modeling of In Vivo Glioma Response After Whole-Brain Radiation Therapy in a Murine Model of Brain Cancer. *Int J Radiat Oncol*. **100** 5 1270–9.

[4] Hormuth II DA, Weis JA, Barnes SL, Miga MI, Rericha EC, Quaranta V, Yankeelov TE. 2017; A

mechanically coupled reaction–diffusion model that incorporates intra-tumoural heterogeneity to predict in vivo glioma growth. *J R Soc Interface*. **14** 128 .

[5] Atuegwu NC, Colvin DC, Loveless ME, Xu L, Gore JC, Yankeelov TE. 2012; Incorporation of diffusion-weighted magnetic resonance imaging data into a simple mathematical model of tumor growth. *Phys Med Biol*. **57** 1 225–40.

[6] Whisenant JG, Ayers GD, Loveless ME, Barnes SL, Colvin DC, Yankeelov TE. 2014; Assessing reproducibility of diffusion-weighted magnetic resonance imaging studies in a murine model of HER2+ breast cancer. *Magn Reson Imaging*. **32** 3 245–9.

[7] Jarrett A, Hormuth II D, Barnes S, Feng X, Huang W, Yankeelov T. 2018; Incorporating drug delivery into an imaging-driven, mechanics-coupled reaction diffusion model for predicting the response of breast cancer to neoadjuvant chemotherapy: theory and preliminary clinical results. *Phys Med Biol*. **63** 10 .

[8] Weis JA, Miga MI, Arlinghaus LR, Li X, Abramson V, Chakravarthy AB, Pendyala P, Yankeelov TE. 2015; Predicting the Response of Breast Cancer to Neoadjuvant Therapy Using a Mechanically Coupled Reaction-Diffusion Model. *Cancer Res*.

[9] Hormuth DA, Jarrett AM, Feng X, Yankeelov TE. 2019; Calibrating a Predictive Model of Tumor Growth and Angiogenesis with Quantitative MRI. *Ann Biomed Eng*. **47** 7 1539–51.

[10] Swanson KR, Rostomily RC, Alvord EC. 2008; A mathematical modelling tool for predicting survival of individual patients following resection of glioblastoma: a proof of principle. *Br J Cancer*. **98** 1 113–9.

[11] Neal ML, Trister AD, Cloke T, Sodt R, Ahn S, Baldock AL, Bridge CA, Lai A, Cloughesy TF, Mrugala MM, et al. 2013; Discriminating Survival Outcomes in Patients with Glioblastoma Using a Simulation-Based, Patient-Specific Response Metric. *PLoS One*. **8** 1 e51951.

[12] Hormuth II D, Eldridge SB, Weis J, Miga MI, Yankeelov TE. 2018. Mechanically Coupled Reaction-Diffusion Model to Predict Glioma Growth: Methodological Details. In: Stechow L von, editor. Springer Methods and Protocols: Cancer Systems Biology. New York, NY: Springer New York; p. 225–41.

[13] Levenberg K. 1944; A method for the solution of certain non-linear problems in least squares. *Q J Appl Mathmatics*. **II** 2 164–8.

[14] Akaike H. 1974. A new look at the statistical model identification. Automatic Control, IEEE Transactions on. p. 716–23.
